# Comprehensive analysis reveals TSEN54 as a robust prognosis biomarker and promising immune-related therapeutic target for hepatocellular carcinoma

**DOI:** 10.18632/aging.204645

**Published:** 2023-04-08

**Authors:** Bidong Fu, Minqin Zhou, Gelin Song, Hong Zeng, Yiyang Gong, Yike Jiang, Yun Ke, Da Huang, Hong Peng, Qing Li

**Affiliations:** 1 Department of Pathology, Second Affiliated Hospital of Nanchang University, Nanchang, Jiangxi Province, People’s Republic of China; 2 Second College of Clinical Medicine, Nanchang University, Nanchang, Jiangxi Province, People’s Republic of China; 3 Department of Thyroid Surgery, Second Affiliated Hospital of Nanchang University, Nanchang, Jiangxi Province, People’s Republic of China; 4 Department of Colorectal Surgery, 908th Hospital of Chinese People’s Liberation Army Joint, Nanchang, Jiangxi Province, People’s Republic of China

**Keywords:** TSEN54, hepatocellular carcinoma, biomarker, immune infiltration, m6A modification

## Abstract

Background: Hepatocellular carcinoma represents the most common primary malignancy of all liver cancer types and its prognosis is usually unsatisfactory. TSEN54 encodes a protein constituting a subunit of the tRNA splicing endonuclease heterotetramer. Previous researches concentrated on the contribution of TSEN54 in pontocerebellar hypoplasia, but no studies have yet reported its role in HCC.

Methods: TIMER, HCCDB, GEPIA, HPA, UALCAN, MEXPRESS, SMART, TargetScan, RNAinter, miRNet, starBase, Kaplan-Meier Plotter, cBioPortal, LinkedOmics, GSEA, TISCH, TISIDB, GeneMANIA, PDB, GSCALite were applied in this research.

Results: We identified the upregulation of TSEN54 expression in HCC and related it to multiple clinicopathological features. Hypomethylation of TSEN54 was closely associated with its high expression. HCC sufferers who held high TSEN54 expression typically had shorter survival expectations. Enrichment analysis showed the involvement of TSEN54 in the cell cycle and metabolic processes. Afterward, we observed that TSEN54 expression level had a positive relationship to the infiltration level of multiple immune cells and the expression of several chemokines. We additionally identified that TSEN54 was related to the expression level of several immune checkpoints and TSEN54 was linked to several m6A-related regulators.

Conclusions: TSEN54 is a prognostic marker of HCC. TSEN54 could become a prospective candidate for HCC diagnosis and therapy.

## INTRODUCTION

Hepatocellular carcinoma (HCC) stands out as the most frequent primary malignancy in all liver cancer types [1]. The morbidity and mortality associated with HCC have increased dramatically in the past decades [2]. Poor progression of HCC patients is also associated with the fact that most HCC cannot be diagnosed in its early stage [3]. Currently, in HCC, early detection can be made by serum biomarker tests (e.g., alpha-fetoprotein [AFP]) and imaging methods (e.g., computed tomography and ultrasound). However, the misdiagnosis rate is high [4–6]. It is widely believed that an accurate and early diagnosis of HCC can significantly improve clinical outcomes and reduce patient suffering. Despite the rapid progress in the discovery of HCC biomarkers, the five-year survival time of patients remains unsatisfactory [7, 8]. Hence, the exploration of valid biomarkers is important for early detection as well as improving outcomes in hepatocellular carcinoma patients.

tRNA Splicing Endonuclease Subunit 54 (TSEN54) is located on 17q25.1 of the human genome and encodes a protein constituting a subunit of the tRNA splicing endonuclease heterotetramer [9]. TSEN54 is a non-catalytic subunit responsible for recognizing the precursor tRNA substrate and for localizing the two catalytic subunits TSEN2 and TSEN34 localize to the 5’ - splice site and 3’ - splice site respectively, thereby splicing the pre-tRNA release intron [10]. Prior researches concentrated on the effect of TSEN54 mutation in pontocerebellar hypoplasia (PCH), with only one report linking it to other RNA processing factors for the construction of a prognostic model of gastric cancer [11–13]. Interestingly, one study used the introns excised during tRNA shearing to generate antisense RNA of cell cycle protein D1, which inhibited the growth of hepatocellular carcinoma cells [14]. This finding attracted us to link TSEN54 with hepatocellular carcinoma and study its role in hepatocellular carcinoma.

The differential expression of TSEN54 between normal tissues and HCC tissues was first evaluated in this study through several public databases and clinical specimens. The correlation of TSEN54 expression with the clinicopathological characteristics of the HCC patients was then explored. Furthermore, we explored the extent of differential promoter methylation of TSEN54 in distinct subgroups. We also constructed the ceRNA regulatory network of TSEN54. Next, statistical methods have been utilized to explore the impact that TSEN54 expression has on survivorship of HCC patients. In addition, a functional enrichment network involving TSEN54 in hepatocellular carcinoma was established. Its involvement in tumor immune cell infiltration, m6A biological processes, and sensitivity to drugs was further dug out. This study reveals TSEN54 as an afresh hepatocellular carcinoma biomarker from several perspectives, which could facilitate diagnosis as well as prognosis assessment and may serve as a therapeutic target.

## MATERIALS AND METHODS

### Data source and processing

This article mainly includes two major databases, The Cancer Genome Atlas (TCGA) database (https://cancergenome.nih.gov) and the International Cancer Genome Consortium (ICGC) database (https://dcc.icgc.org/projects/LIRI-JP). To date, TCGA has published data regarding 33 types of cancers, and our study researched TSEN54 expression in HCC using 374 tumor samples and 50 normal samples in the TCGA database with file type HTSeq-FPKM [15]. Other than this, through 377 clinical samples in TCGA, we drilled down the clinicopathological features of TSEN54 in HCC and the impact of TSEN54 on the survivorship of HCC patients. Moreover, we also made use of data from the ICGC [LINC-JP] Liver Cancer– NCC to verify the above conclusion, which involved 202 normal samples and 243 tumor samples.

### TIMER database analysis

TIMER (https://cistrome.shinyapps.io/timer/) is a synthetic network that analyzes the immune invasion level in different cancers [16]. We used “Diff Exp” to identify the expression of TSEN54 in multiple tumors. “Genes” and “SCNA” were then applied to estimate the relation between tumor infiltrating-immune cells and the expression of mRNA and copy number variations of TSEN54 in HCC. Finally, we verified the relevance between TSEN54 expression and immune cell markers and immune checkpoints in HCC with “correlation”.

### HCCDB database analysis

The Hepatocellular Cancer Database (HCCDB) (http://lifeome.net/database/hccdb/home.html) is a database dedicated to hepatocellular carcinoma, and it contains 15 publicly available expression datasets about HCC, altogether 3917 samples [17]. Using this database, we investigated the TSEN54 expression in HCC tissues and adjacent tissues in 10 datasets.

### GEPIA database analysis

The Gene Expression Profiling Interactive Analysis (GEPIA) database (http://gepia.cancer-pku.cn/) uses data from TCGA and GTEx to present subscribers with customized functionality [18]. Together with the data from TCGA, we employed it to draw box plots for the analyses of TSEN54 expression in HCC and normal tissues. Besides, we exploited both Overall Survival (OS) and Disease-Free Survival (RFS) survival plots for survival analysis of TSEN54 in HCC patients and examined the impact of m6A-related regulators on the prognosis of HCC patients by OS curves.

### HPA analysis

The Human Protein Atlas (HPA) (https://www.proteinatlas.org/) is a versatile database that analyzes gene expression in human proteins from different aspects [19]. From the HPA database, we obtained the outcome of staining tissues from diverse patients to verify the protein expression level of TSEN54 in normal tissues (Patient id:3402) and HCC tissues (Patient id:3477).

### UALCAN database analysis

The UALCAN database (http://ualcan.path.uab.edu) is an interactive portal online tool that delves into TCGA gene expression data and can be applied to research multiple cancers [20]. In our research, we explored the clinicopathological features, methylation and survival of TSEN54 in HCC via it.

### cBioPortal analysis

cBioPortal (https://www.cbioportal.org/) is an integrated web resource, presenting us with visual and multidimensional cancer genomics data [21]. Based on Liver Hepatocellular Carcinoma (TCGA, Firehose Legacy) dataset, we monitored TSEN54 methylation level linked to its mRNA expression.

### MEXPRESS analysis

MEXPRESS (https://mexpress.be) online website is used to visualize DNA methylation and gene expression, as well as the correlation between them [22]. We studied the relationship between the methylation and expression level of some TSEN54 CpG sites by it.

### SMART analysis

Shiny methylation analysis resource tool (SMART; http://www.bioinfo-zs.com/smartapp/) is a site which is friendly to subscribers, and it can be applied to evaluate DNA methylation data from the TCGA database [23]. By searching “TSEN54,” we researched the allocation of methylation probes associated with TSEN54 on the chromosome.

### Construction of ceRNA regulatory network

The TargetScan database (http://www.targetscan.org) allows for the prediction of probable miRNA binding sites and contributes to the placement of miRNAs into gene regulatory networks [24]. RNAinter (http://www.rnainter.org) online site incorporates experimentally confirmed and computationally forecasted RNA interactome data, which facilitates us to study the regulatory environment of cellular RNA [25]. Our study extracted the data from these two databases to take the intersection for the purpose of screening out the target miRNAs of TSEN54. miRNet 2.0 (https://www.mirnet.ca/miRNet/home.xhtml) is a database that integrates user data with existing knowledge for the clarification of miRNAs [26]. starBase (https://starbase.sysu.edu.cn/) online website available for the decoding of RNA-RNA and protein-RNA interaction networks [27]. We exploited both two platforms to identify target lncRNAs for hsa-miR-125b-5p and hsa-miR-27b-3p.

### Kaplan-Meier plotter database analysis

Kaplan-Meier Plotter database (http://kmplot.com) is available to measure the connection between gene expression and survivorship, whose data is derived from GEO, EGA and TCGA databases [28]. Separating TSEN54 expression into high and low groups, our study not only inspected the relevance of TSEN54 expression to overall survival (OS), progression-free survival (PFS), relapse-free survival (RFS) and disease-specific survival (DSS) in HCC, but also explored the immune cells affecting HCC patients‘ survival.

### LinkedOmics analysis

LinkedOmics database (http://linkedomics.org/login.php) can be applied to discuss the co-expression of genes and their corresponding functional enrichment analysis [29]. Our study used the LinkFinder module to structure a volcano map and heatmaps to illustrate the co-expression of TSEN54 genes and displayed their correlation by plotting scatter plots. The LinkInterpreter module was applied to establish Go annotations and KEGG pathways with the purpose of describing the pathways through which these genes function. The Spearman correlation test in this study is our statistical method.

### Gene set enrichment analysis (GSEA)

GSEA can explain gene expression data by testing if the gene set was enriched at the top or bottom of a pre-set sequencing table [30]. Our research divided the TCGA-LIHC data into two groups with TSEN54 high and low expression to study the possible influence of the TSEN54 gene set on HCC pathways via GSEA v4.2.2. We configure some parameters as follows: gene set database: kegg. v7.5.1 symbols. gmt (Curated), meanwhile, *P* < 0.05 and *FDR* < 0.25 were regarded meaningful.

### TISCH analysis

The Tumor Immune Single Cell Center (TISCH) (http://tisch.comp-genomics.org/) database is concentrating on the examination of the tumor microenvironment, gathering data from the Gene Expression Omnibus (GEO) and ArrayExpress for 27 cancer types and 76 tumor datasets [31]. It was utilized in our study for the visualization of the single-cell level expression status of TSEN54 in two immune cell datasets, which were LIHC_GSE140228_10× and LIHC_GSE140228_Smartseq2.

### TISIDB analysis

TISIDB (http://cis.hku.hk/TISIDB/) is a comprehensive database could be employed to analyze interactivity between neoplasms and the immune system [32]. We predicted the prognosis of HCC patients and explored TSEN54 expression in diverse immune subtypes, as well as the relationship of TSEN54 expression with chemokines in HCC utilizing the TISIDB database.

### Quantitative real-time PCR

The HCCLM3 cell line was obtained from the Cell Bank of Type Culture Collection of the Chinese Academy of Sciences and the Shanghai Institute of Cell Biology in China. We extracted total RNA from cell samples applying Trizol reagent (Thermo Fisher Scientific) by following the manufacturer’s guidelines, then performing reverse transcription reactions employing the PrimeScript RT Reagent Kit (Invitrogen, USA). And the qPCR was completed utilizing SYBR Premix Ex Taq (TaKaRa, China) [33]. This technique was adopted in this study to examine the relative mRNA expression of CCL15, CCL26, and CCL28.

### GeneMANIA analysis

GeneMANIA (https://genemania.org/) database can be used to find genes that share functions with the gene of interest and to explore interactions and functions between them [34]. Through GeneMANIA database, we researched the connection between TSEN54 and other related genes.

### Protein structure and docking analysis

The secondary structures of TSEN54 and its physically interacting proteins were acquired from the “Mutations” panel in the cBioPortal database (https://www.cbioportal.org/) for the dataset: Liver Hepatocellular Carcinoma (TCGA, Firehose Legacy) [35]. CSNK2B and ATM advanced structures were acquired by using the PDB database (https://www.rcsb.org/) (PDB ID: 3EED and 7SIC) [36]. The advanced structure of TSEN54 was predicted by AlphaFold Protein Structure Database (https://www.alphafold.ebi.ac.uk/) (UniProt ID: Q7Z6J9) [37]. Finally, the interaction docking patterns between TSEN54 and CSNK2B as well as TSEN54 and ATM were predicted by the HDOCK server (http://hdock.phys.hust.edu.cn/) and visualized using PyMOL software [38].

### GSCALite analysis

Gene Set Cancer Analysis (GSCALite) (http://bioinfo.life.hust.edu.cn/web/GSCALite/) integrates information from TCGA, Drug Sensitivity in Cancer (GDSC), Cancer Therapeutics Response Portal (CTRP), GTEx (Genotype-Tissue Expression) to users [39]. Our research utilized data from TCGA-LIHC and GTEx_Liver to explore TSEN54-related cancer pathways and analyzed drug susceptibility in HCC by GDSC.

### Statistical analysis

Our article performs all data analysis by R software (version 3.6.3/4.1.2). We adopted the rank-sum test to detect the expression of TSEN54 in HCC tissues and normal tissues according to the R packages “limma” and “beeswarm”, and the statistical methods of the Wilcox. test and Kruskal. test and logistic regression analysis were applied to probe the link between TSEN54 and clinicopathological characteristics. What is more, for the purpose of examining the connection between TSEN54 expression and the outcome of HCC sufferers, we drew Kaplan-Meier survival curves by log-rank test and created a univariate and multivariate Cox regression model through the R packages “survival” and “survminer” (*P* < 0.05), and we also built ROC curves by “survival ROC” to assess the accuracy of TSEN54 expression in predicting the survival rate of HCC patients at 1, 3 and 5 years.

### Data availability

The TCGA data used to support the findings of this study have been deposited in the [https://cancergenome.nih.gov] repository (doi:10.1038/nmeth.2956).

## RESULTS

### TSEN54 expression is upregulated in HCC

**Figure 1:**
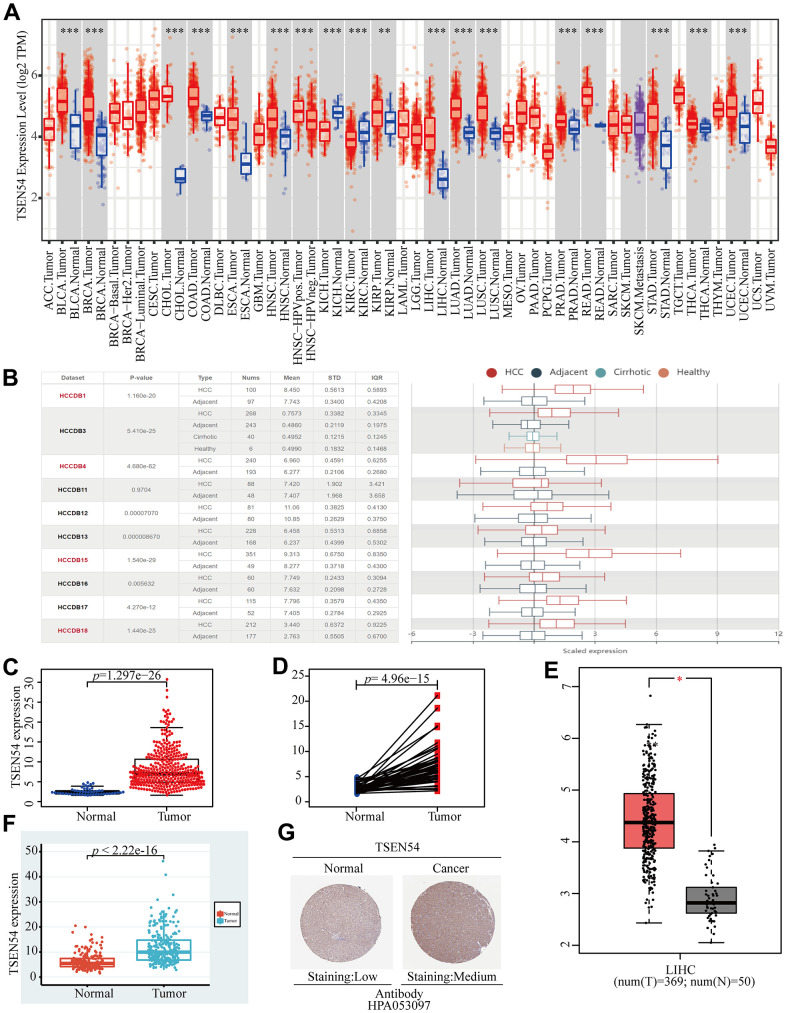
**The expression analysis of TSEN54 in HCC.** (**A**) Differential expression of TSEN54 in pan-carcinoma and corresponding normal tissues in TIMER. (**B**) Charts and graphs present the TSEN54 expression in HCC and normal tissues downloaded from the HCCDB database. (**C**) Plots describe the mRNA expression level of TSEN54 in normal and HCC samples. (**D**) Paired differential expression of TSEN54 in normal and HCC tissues. The difference representation of TSEN54 in HCC and normal samples was plotted using the (**E**) GEPIA website and the (**F**) ICGC database. (**G**) IHC staining results show the protein expression level of TSEN54 in liver normal tissues and tumor tissues.

With the purpose of assessing TSEN54 differential expression in HCC and normal samples, we attempted a variety of methods. In the beginning, we made a study of the TSEN54 expression level in pan-cancer on the TIMER database. The result demonstrated that the expression of TSEN54 was remarkably higher in multiple cancer tissues involving HCC in comparison with normal tissues (Figure 1A). Then, applying the HCCDB database to verify the above result, we also discovered that the finding was in line with our expectations (Figure 1B). After that, we further investigated the expression of TSEN54 via data from TCGA depending on “limma” and “beeswarm” packages from R software, and TSEN54 expression was detected meaningfully higher in HCC tissues than in normal tissues (Figure 1C). Moreover, we made a comparison of the TSEN54 expression of HCC between 50 pairs of tumor samples and normal samples, the same outcome was obtained (Figure 1D). What is more, as exhibited in Figure 1E, 1F, we still consistently ascertained identical results in both the GEPIA website and ICGC database. Ultimately, we obtained tissue slices through the HPA database to further corroborate our conclusion, and it can be observed from the IHC staining results that the TSEN54 protein expression in HCC tissues displayed higher levels versus normal liver tissues (Figure 1G). Above all, we can come to the conclusion that in contrast to normal tissues, the TSEN54 expression in HCC tumor tissues is upregulated.

### The correlation between the expression of TSEN54 and clinicopathological features

**Figure 2:**
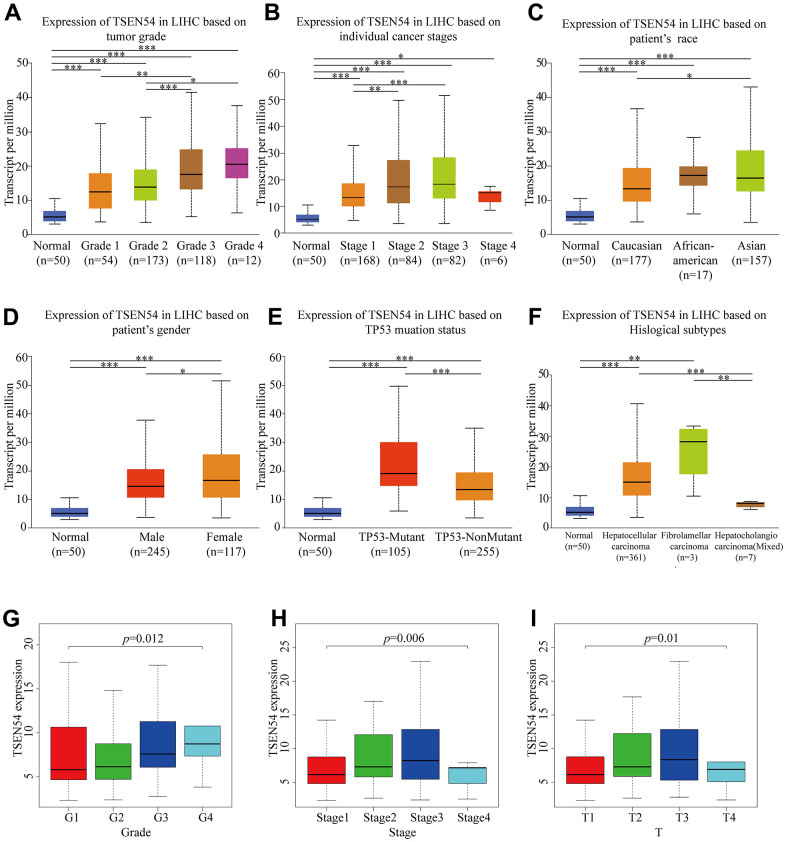
**TSEN54 expression is associated with multiple clinicopathological features.** Box-whisker plots reveal the variability of gene expression level ground on subgroup (**A**) tumor grade; (**B**) individual cancer stages; (**C**) patient’s race; (**D**) patient’s gender; (**E**) TP53 mutation status; (**F**) histological subtypes. The relationship between TSEN54 expression and (**G**) grade; (**H**) stage; (**I**) T stage in HCC according to data from the TCGA database. (*, *p* < 0.05; **, *p* < 0.01; ***, *p* < 0.001).

Digging out the connection between the expression of TSEN54 in HCC and clinical pathological features is of great practical importance for our research. Through TCGA-LIHC samples from the UALCAN database, we conducted a subgroup analysis of several clinical pathological characteristics. According to Figure 2A–2F, we did know that TSEN54 was related to the grade, stage, race, gender, TP53 mutation status, and histological subtypes in HCC. Interestingly, we also observed that the expression of TSEN54 in grade1 and stage1 had a certain degree of increase in HCC compared to normal, and it was also very significant between many other subgroups of grade and stage (Figure 2A, 2B). Next, we downloaded clinical samples from the TCGA database and then made use of the Wilcox. test and Kruskal. test in R software, again finding that TSEN54 expression in HCC was highly related to grade (p=0.012), stage (p=0.006) and T (p=0.01) (Figure 2G–2I). Lastly, we further verified our conclusion by logistic regression analysis and uncovered it to be consistent with our previously stated consequence (Supplementary Table 1). All findings point to an inextricable relationship between TSEN54 expression in HCC and multiple clinicopathological features.

### High expression of TSEN54 is affected by hypomethylation

**Figure 3:**
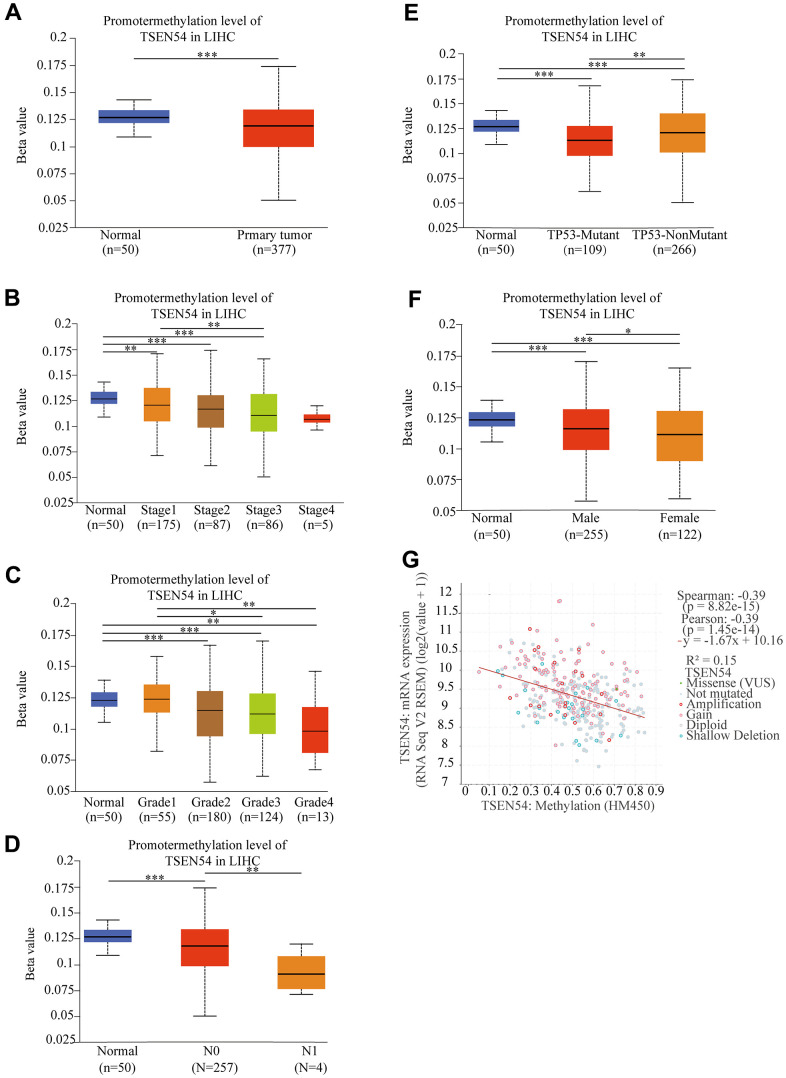
**The TSEN54 promoter methylation level is correlated with clinicopathological features.** (**A**) sample type; (**B**) stage; (**C**) grade; (**D**) lymph node metastasis; (**E**) TP53 mutation status; (**F**) patient’s gender. (**G**) The correlation between TSEN54 methylation and its mRNA expression.

There is growing evidence that illustrates methylation has a crucial part in cancer emergence and progression [40]. Firstly, we applied the UALCAN website and revealed that TSEN54 promoter methylation level in HCC tissues were lower than those in normal samples (Figure 3A). Additionally, we identified that the TSEN54 promoter methylation level was linked to clinical stage, histological grade and lymph node metastasis (Figure 3B–3D). Moreover, HCC patients with TP53 mutations have lower methylation level compared to those without mutations (Figure 3E). Besides, in the sex subgroup of patients, TSEN54 methylation level in the HCC tissues of female patients were lower than those of male patients (Figure 3F). The subtle contrast in clinicopathological features between the methylation results and the expression results in Figure 2 impelled us to directly detect the relation between mRNA expression and methylation of TSEN54 using the cBioPortal website, and unsurprisingly, the two behave as a negative correlation (Figure 3G).

**Figure 4:**
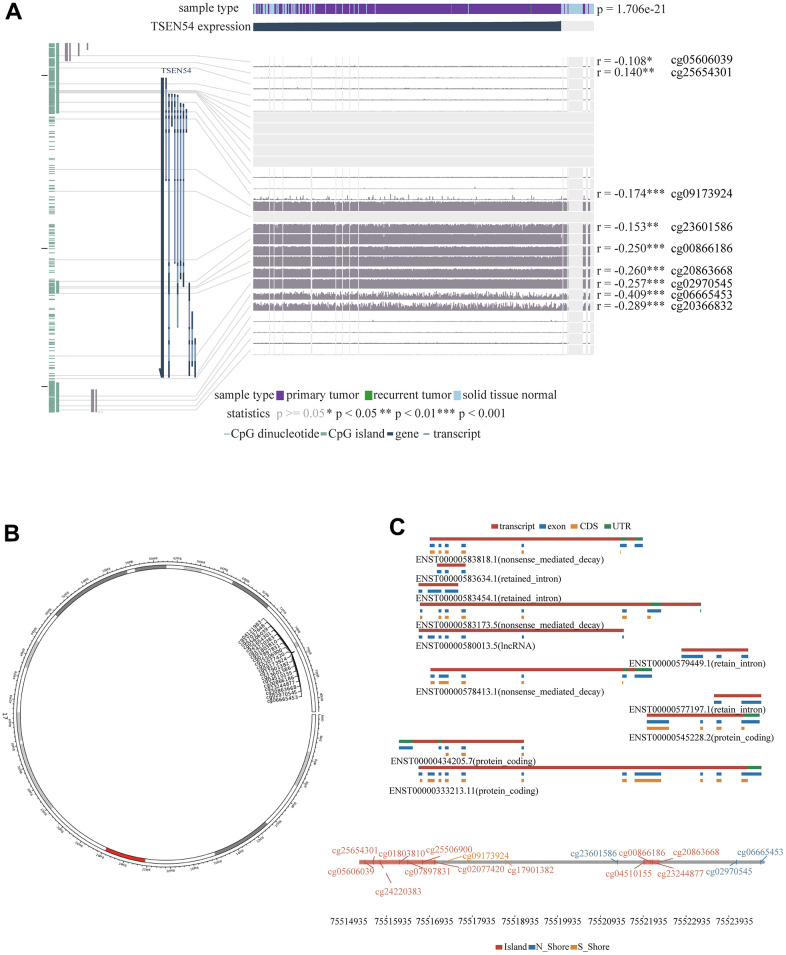
**Distribution of CpG sites on TSEN54.** (**A**) Correlation between TSEN54 expression and methylation of the CpG sites. (**B**) Distribution of TSEN54 methylation probes on chromosome. (**C**) Location of CpG sites associated with TSEN54.

Then, through the MEXPRESS online website, we found that the methylation level of 9 CpG sites were correlated with TSEN54 expression. Among them, the methylation level of 8 CpG sites including cg05606039, cg09173924, cg23601586, cg00866186, cg20863668, cg02970545, cg06665453, cg20366832 were negatively associated with TSEN54 expression level (Figure 4A). Furthermore, we utilized the SMART website to lucubrate the distribution of methylation probes related to TSEN54 on chromosome (Figure 4B). The result shown that cg05606039, cg00866186, and cg20863668 are on the island, cg02970545, cg06665453, and cg23601586 are on N_Shore, and cg09173924 is on S_Shore (Figure 4C). In conclusion, we discovered hypomethylation of TSEN54 in HCC, which contributes to its high expression.

### The construction of a ceRNA regulatory network of TSEN54 in HCC

**Figure 5:**
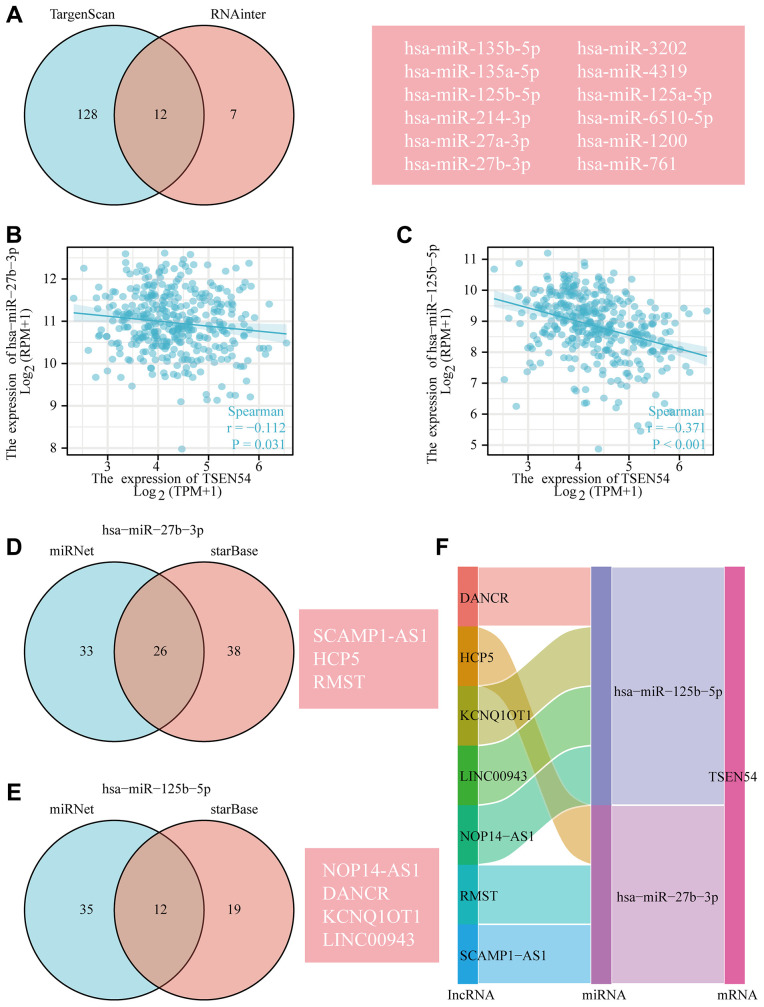
**The predicted ceRNA network of TSEN54 in HCC.** (**A**) The Venn diagram demonstrates the miRNAs targeting TSEN54 predicted by TargetScan and RNAinter databases. The correlation scatter plots reveal the expression relevance of TSEN54 with (**B**) hsa-miR-27b-3p and (**C**) hsa-miR-125b-5p. Venn diagram illustrates the prediction of lncRNAs targeting (**D**) hsa-miR-27b-3p and (**E**) hsa-miR-125b-5p through miRNet and starBase tools. (**F**) Sankey diagram presents the ceRNA network of TSEN54 in HCC.

In an attempt to better gain an understanding of the possible regulatory mechanisms at the level of TSEN54 expression, we predict and build a lncRNA-miRNA-mRNA ceRNA network that involves TSEN54 in HCC employing multiple bioinformatics databases. Firstly, we jointly predicted 12 miRNAs targeting TSEN54 by TargetScan and RNAinter databases, which were hsa-miR-135b-5p, hsa-miR-135a-5p, hsa-miR-125b-5p, hsa-miR-214-3p, hsa-miR-27a-3p, hsa-miR-27b-3p, hsa-miR-3202, hsa-miR-4319, hsa-miR-125a-5p, hsa-miR-6510-5p, hsa-miR-1200, hsa-miR-761 (Figure 5A). Since miRNAs usually have the capacity to negatively regulate mRNA, two miRNAs (hsa-miR-27b-3p and hsa-miR-125b-5p) that were negatively relevant to TSEN54 at the expression level were filtered from those 12 miRNAs, and their correlation scatter plots were given in Figure 5B, 5C. Afterward, utilizing miRNet and starBase tools, a series of 26 lncRNAs that may bind to hsa-miR-27b-3p and 12 lncRNAs that combine with hsa-miR-125b-5p were further predicted (Figure 5D, 5E). Similarly, as lncRNAs are usually able to negatively regulate miRNAs, we continued to screen for lncRNAs that correspond to a negative association with the two miRNAs at the expression level. From our findings, for hsa-miR-27b-3p, the eligible lncRNAs were SCAMP1-AS1, HCP5, and RMST (Figure 5D); while for hsa-miR-125b-5p, NOP14-AS1, DANCR, KCNQ1OT1 and LINC00943 were qualified (Figure 5E). In the end, based on the hypothesis that there is an inverse relationship between miRNA and lncRNA or mRNA, we constructed a ceRNA regulatory network, and the results are exhibited in the Sankey diagram (Figure 5F).

### High expression of TSEN54 is a separate factor for poor prognosis in HCC patients

**Figure 6:**
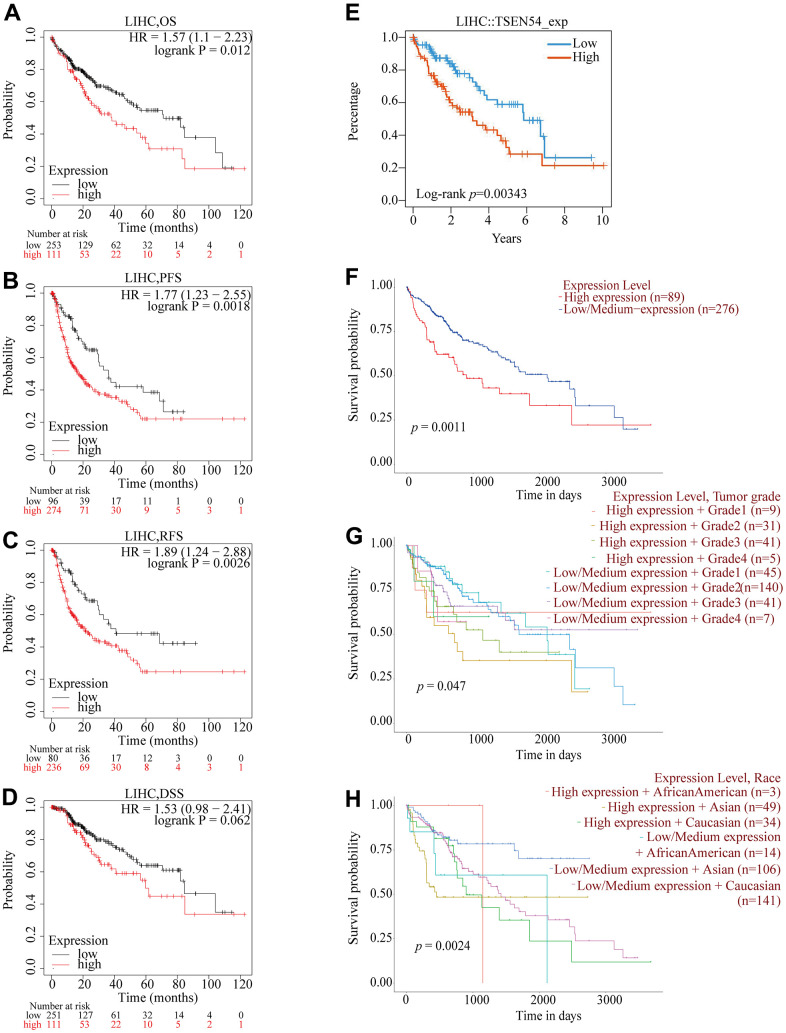
**Relationship between TSEN54 expression and prognosis of HCC patients.** The relation between TSEN54 expression and (**A**) OS; (**B**) PFS; (**C**) RFS; (**D**) DSS based on the Kaplan-Meier plotter database. (**E**) The correlation between TSEN54 expression and the survival of HCC patients based on the TISIDB website. Survival of HCC patients with (**F**) differential TSEN54 expression; (**G**) differential TSEN54 expression and grade; (**H**) differential TSEN54 expression and race.

For the sake of analyzing the prognostic value of TSEN54 expression in HCC patients, we made use of several online websites. The association between TSEN54 expression and prognosis of HCC patients was initially investigated using the GEPIA website. In LIHC datasets, patients with high TSEN54 expression had shorter OS and DFS compared to those with low expression (Supplementary Figure 1). Next, the Kaplan-Meier Plotter site indicated that HCC suffers with higher TSEN54 expression possessed poorer OS, PFS and RFS, while the differential expression of TSEN54 in the DSS group had no significant impact on survival (Figure 6A–6D). Besides, an analogous outcome was likewise backed up by the TISIDB website (Figure 6E). Deepening, we hired the UALCAN website and identified that not only can the differential expression of TSEN54 impinge on patient prognosis, but TSEN54 expression combined with clinicopathological features (e.g., grade and race) can also affect the survival of HCC patients (Figure 6F–6H).

**Figure 7:**
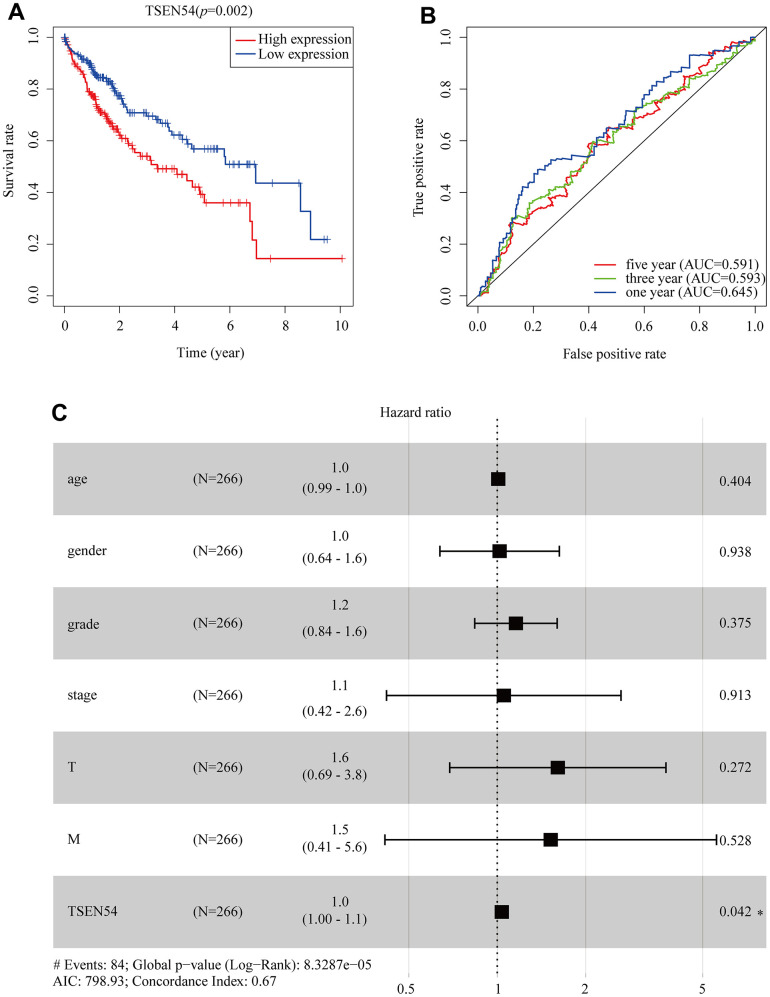
**TSEN54 is an independent prognostic factor for the survival of HCC patients.** (**A**) The association between TSEN54 expression and HCC patients based on data downloaded from TCGA. (**B**) ROC curve for one-, three-, and five-year survival according to TSEN54 expression level. (**C**) The forest plot displays the result of the multivariate analysis. *p< 0.05; **p< 0.01; ***p< 0.001.

In parallel, we optimized patient survival data collected from the TCGA database and again demonstrated a shorter prognosis within the TSEN54 expression upregulated group (Figure 7A). Thereafter, the ROC curve was created through the TCGA data to reflect the prognostic value of TSEN54. The AUC of the ROC curve was significant (one year AUC: 0.645, three years AUC: 0.593, five years AUC: 0.591), indicating that the expression of TSEN54 was a valid predictor for prognosis in HCC patients (Figure 7B). Eventually, the connection between clinicopathological features and the prognosis of patient data extracted from the TCGA database was calculated by constructing univariate and multivariate cox models. Univariate analysis revealed a close correlation between stage, T stage, M stage, TSEN54 expression, and deterioration of OS. Multivariate analysis demonstrated TSEN54 expression to be a separate prognostic factor (Supplementary Table 2). This is directly reflected in the forest map (Figure 7C). In conclusion, the expression of TSEN54 is a standalone prognostic biomarker of HCC patients, while hyper-expression of TSEN54 is linked to the unfavorable prognosis of HCC patients.

### Co-expression network of TSEN54 and its functional enrichment analysis

**Figure 8:**
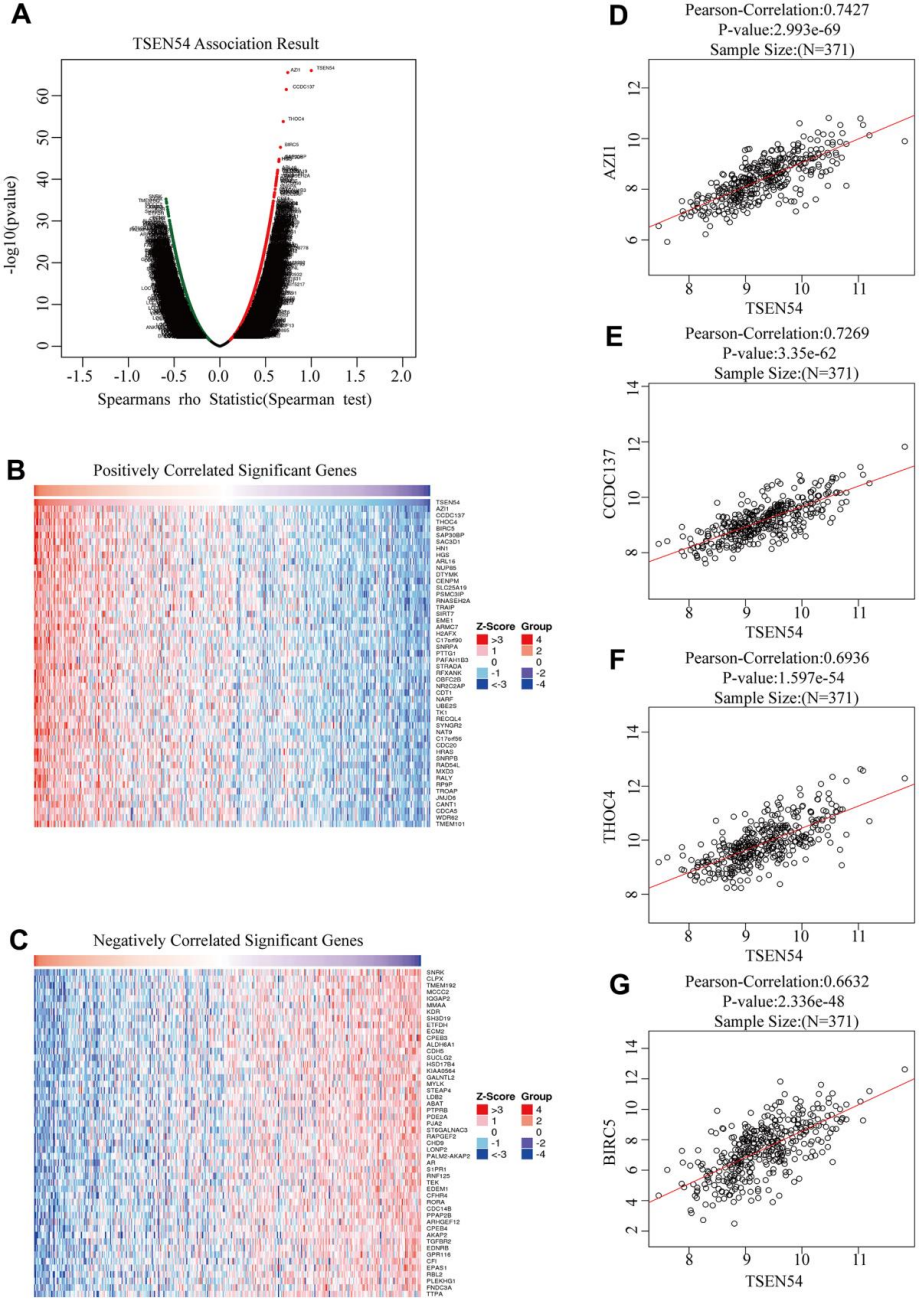
**Co-expressed genes of TSEN54 in HCC (LinkedOmics).** (**A**) The volcano map reveals the positive and negative co-expression genes of TSEN54 and roughly illustrates the correlation between them. Heatmaps show the first fifty genes most (**B**) positively and (**C**) negatively linked to TSEN54. (**D**–**G**) The scatter plots detail the link between TSEN54 and its hub genes. GO: Gene Ontology; KEGG: Kyoto Encyclopedia of Genes and Genomes.

**Figure 9:**
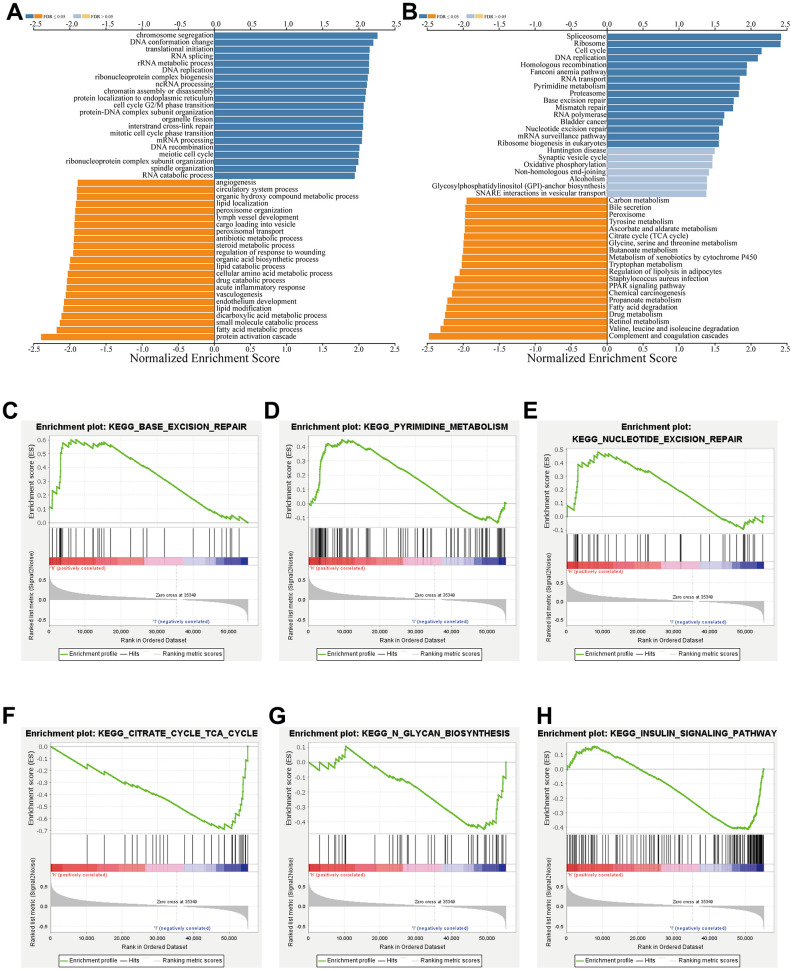
**Functional enrichment study of TSEN54 co-expressed genes.** (**A**) Go annotations and (**B**) KEGG pathways with a notable enrichment of the TSEN54 co-expression genes in HCC by LinkedOmics. (**C**–**H**) Analysis of the respective pathways enriched in high and low expression of TSEN54 and its co-expressed genes according to GSEA.

To detect co-expressed genes of TSEN54 and probe their biological functions in HCC, we did a series of works. We first utilized the LinkedOmics database to investigate the co-expression genes of TSEN54. As indicated in the volcano map, red dots represented genes positively associated with TSEN54, totaling 6,679, while green dots meant genes negatively correlating with the target genes, amounting to 5,702 (Figure 8A). The 50 genes most strongly positively and negatively related to TSEN54 were exhibited in heatmaps (Figure 8B, 8C). In the following, we opted the four genes that were most prominently positively correlated with TSEN54 called the hub genes to create scatter plots, which were AZI1, CCDC137, THOC4, and BIRC5 (Figure 8D–8G). Subsequently, we made a deeper exploration of the potential functions of TSEN54 and its co-expressed genes in HCC. From the GO outcome, we could conclude that TSEN54 was mainly concentrated in the pathways like chromosome segregation, DNA replication, cell cycle G2/M phase transition, interstrand cross-link repair, RNA catabolic process, etc. (Figure 9A). Meanwhile, the KEGG findings as revealed in Figure 9B suggested that TSEN54 may function through spliceosome, ribosome, cell cycle, DNA replication, homologous recombination, Fanconi anemia pathway, pyrimidine metabolism, PPAR signaling pathway, etc.

Additionally, we made further investigation based on GSEA to probe the pathways by which TSEN54 and some of its relative genes may exert influence on HCC. The findings implied that it may have a connection with DNA repair and metabolism-related pathways, such as base excision repair, pyrimidine metabolism, nucleotide excision repair and insulin signaling pathway (Figure 9C–9H). From the outcomes mentioned above, we draw a conclusion that the expression of TSEN54 may function critically in HCC by modulating the cell cycle, substance metabolism and DNA repair.

### Expression of TSEN54 in immune cells of the tumor microenvironment

**Figure 10:**
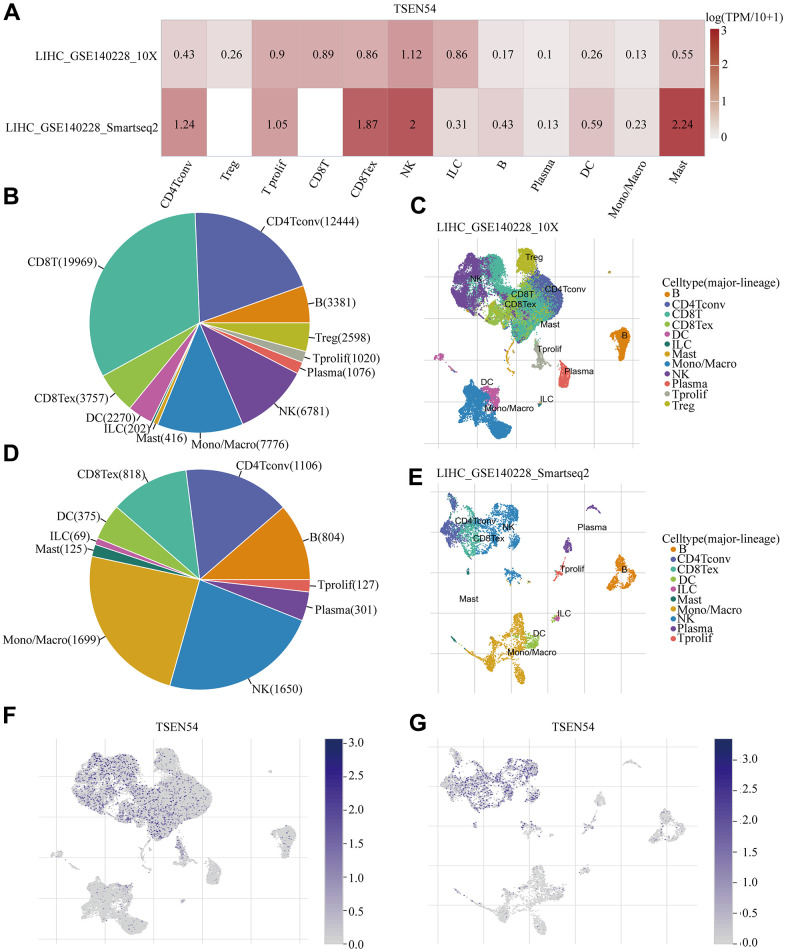
**Expression of TSEN54 in HCC at the single cell level.** (**A**) Heatmap reveals the expression level of TSEN54 in a variety of immune cells on LIHC_GSE140228_10× and LIHC_GSE140228_Smartseq2 datasets. (**B**–**E**) Types and distribution of cells in the LIHC_GSE140228_10× and LIHC_GSE140228_Smartseq2 datasets. The t-SNE plots describe the distribution of TSEN54 expression in (**F**) LIHC_GSE140228_10× and (**G**) LIHC_GSE140228_Smartseq2 datasets of individual cell clusters.

Aiming to characterize TSEN54 expression in tumor microenvironment immune cells at the single cell level, we drew on two-single cell sequencing datasets from the TISCH database, which were LIHC_GSE140228_10× and LIHC_GSE140228_Smartseq2. We noticed that the expression of TSEN54 translated by log (TPM/10+1) manifested heterogeneity among distinct cell clusters with respect to diverse datasets, and the highest expression level of TSEN54 was evident in Mast cells of the LIHC_GSE140228_Smartseq2 dataset (Figure 10A). Later on, we had further exploration of two datasets, the results were as follows: the LIHC_GSE140228_10× dataset was classified into 12 cell types, of which the CD8T cell count was the most abundant (19969) (Figure 10B), plus the distribution and quantity of varied immune cells were described in Figure 10C; Also, Figure 10D consisted of 10 cell types from the LIHC_GSE140228_Smartseq2 dataset, whose distribution was given in Figure 10E, and it was noted that Mono/Macro cell and NK cell had the largest and similar numbers of cell count, with the respective count of 1699 and 1650. Additionally, the distribution of TSEN54 gained employing single-cell resolution for all kinds of immune cells in the LIHC_GSE140228_10× and LIHC_GSE140228_Smartseq2 datasets were plotted in Figure 10F, 10G, which were consistent with the finding in Figure 10A. In summary, we are able to discover that TSEN54 is expressed in almost all immune cells, especially NK cells and Mast cells by single-cell sequencing.

### Association between TSEN54 expression and immune infiltration in HCC

**Figure 11:**
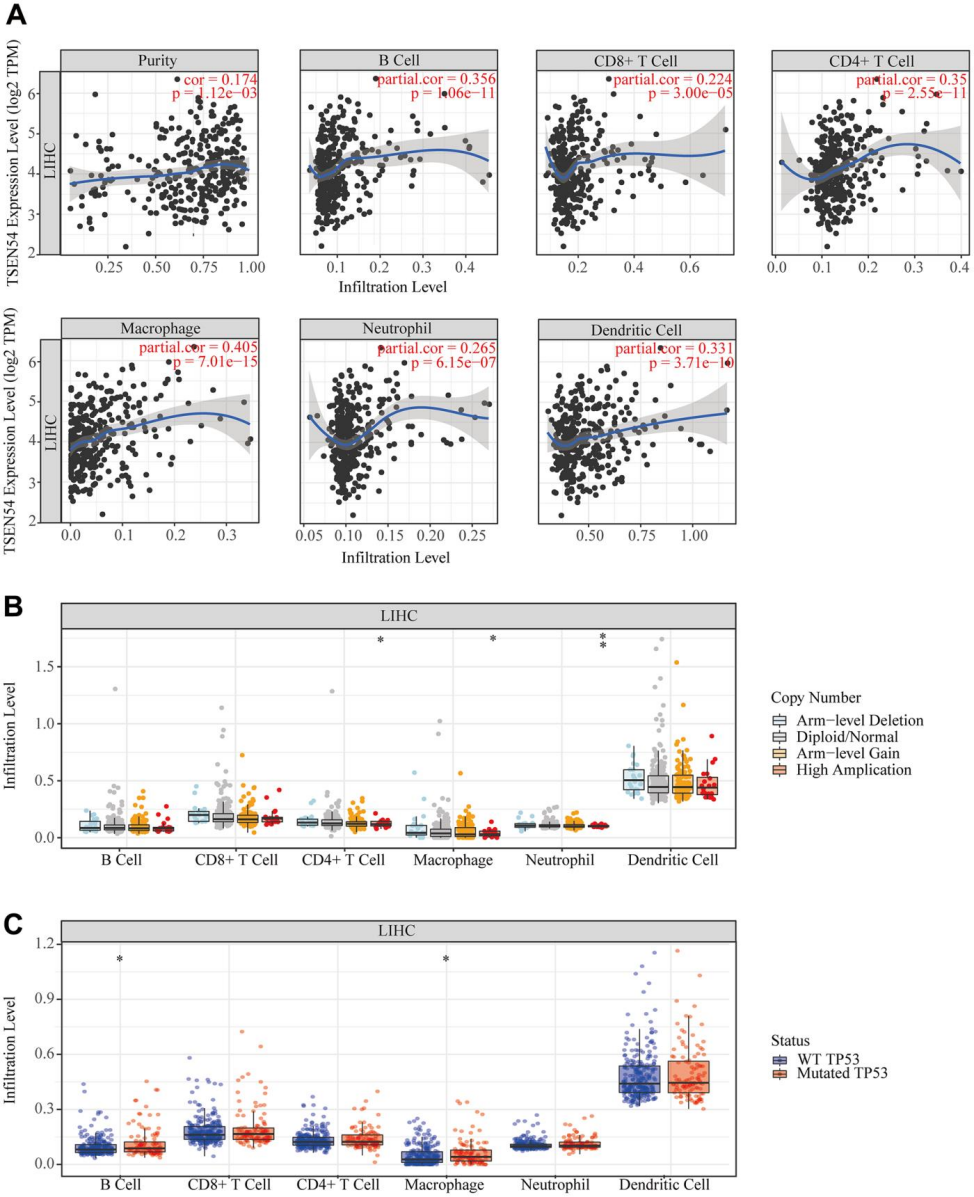
**The relationship between TSEN54 expression and immune infiltration in hepatocellular carcinoma.** (**A**) The correlation between the immune cell infiltration level and the expression of TSEN54. (**B**) The connection between the CNV of TSEN54 and the immune cell infiltration level. (**C**) The relation between the TP53 mutation status and the immune cell infiltration level.

Since there is a strong association between immunological characteristics of cancer and prognosis, the relation between TSEN54 expression and immune cell infiltration was first investigated utilizing the TIMER database. Consequences indicated that the TSEN54 expression level in HCC was positively correlated with B cells, CD8+T cells, CD4+T cells, macrophages, neutrophils, and dendritic cells infiltration level (Figure 11A). Then we investigated the copy number variation (CNV) of TSEN54 in relation to immune cell infiltration level. Inexplicably, high amplification of TSEN54 had lower level of infiltration of CD4+ T cells, macrophages and neutrophils (Figure 11B). To delve into the explanations between TSEN54 expression and immune cell infiltration, we examined the linkage between TSEN54 expression and relevant immune cell markers. The correlation coefficient was regulated by tumor purity. The outcomes demonstrated a positive correspondence between the TSEN54 expression and a multitude of immune cell markers (Supplementary Figure 2) (Supplementary Tables 3, 4). Separately we noticed a higher level of B cell and macrophage infiltration in the TP53 mutant group versus the wild-type TP53 subgroup (Figure 11C).

### Relationship between TSEN54 expression and immune subtype and chemokine expression in HCC

Later, we researched the association between TSEN54 expression and different immune subtypes and immune chemokines expression in HCC by TISIDB. It was found that the expression of TSEN54 was higher in C1 (would healing) and C2 (IFN-gamma dominant) subtypes, but was lower in C3 (inflammatory) and C6 (TGF-B dominant) (Supplementary Figure 3A). The expression level of chemokines XCL1, CCL15, CCL20, CCL26, CCL28, and TSEN54 were positively correlated (Supplementary Figure 3B–3F). In addition, our assay of chemokines expression in HCCLM3 cells with different treatments demonstrated that the expression of CCL15, CCL26, and CCL28 was downregulated in shTSEN54 cells (Supplementary Figure 3G–3I).

### Role of TSEN54 in the regulation of immune response to HCC

Aiming to study the action of TSEN54 in the immune response, we again exploited the TIMER website to conduct the relation between the expression of TSEN54 and common immune checkpoints. The consequence reflected that TSEN54 expression was positively linked to most of the expression of immune checkpoints, especially CD276 (B7-H3), CTLA4, HAVCR2, and PDCD1 (PD-1) (Supplementary Figure 4). This outcome implied a possible involvement of TSEN54 in suppressing the immune response and helping tumors to undergo immune escape.

### The prognostic impact of TSEN54 expression is related to immune infiltration

**Figure 12:**
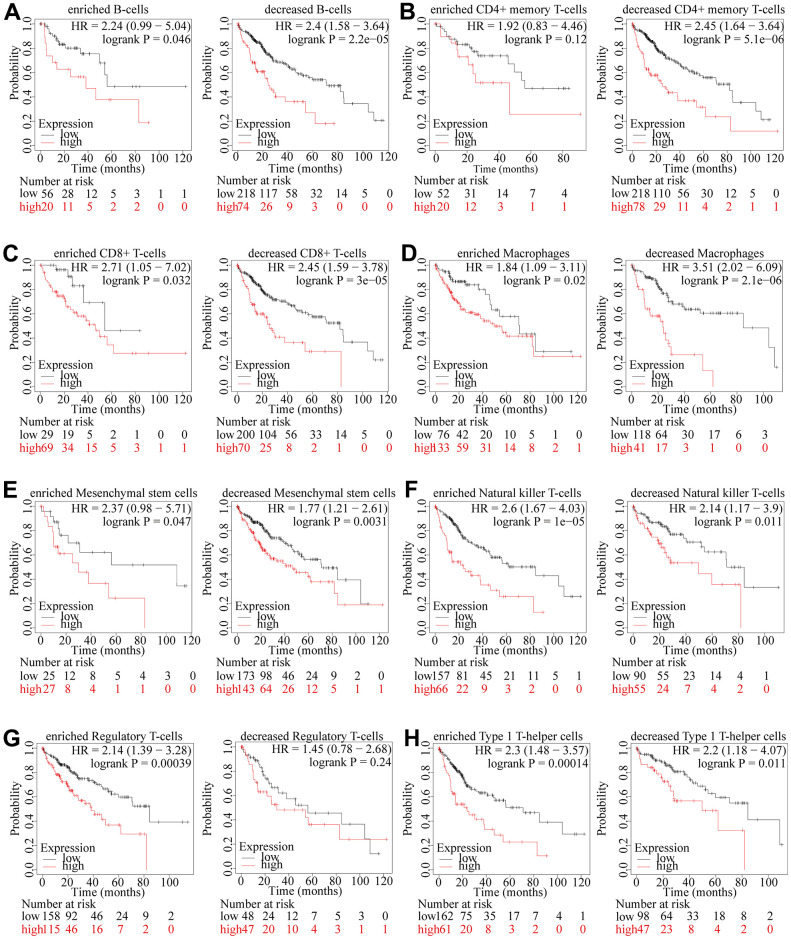
**The prognostic influence of TSEN54 expression in HCC is linked to the infiltration level of immune cells.** (**A**) B cells; (**B**) CD4+ memory T-cells; (**C**) CD8+ T-cells; (**D**) Macrophages; (**E**) Mesenchymal stem cells; (**F**) Natural killer T-cells; (**G**) Regulatory T-cells; (**H**) Type 1 T-helper cells. *p < 0.05, **p < 0.01, ***p < 0.001.

Since TSEN54 expression was remarkably associated with immune infiltration in HCC, we then utilized the Kaplan-Meier Plotter website to explore whether the prognostic impact of TSEN54 expression was also associated with immune infiltration. As it turns out, the poor prognostic ending of patients with high TSEN54 expression was not altered irrespective of the enrichment or decrease of B cells, CD8+ T cells, macrophages, mesenchymal stem cells, natural killer T cells and type 1 T helper cells (Figure 12A, 12C–12F, 12H). Excitingly, when CD4+ memory T-cells were enriched, changes in TSEN54 expression could no longer impinge on survival (Figure 12B). Similarly, when regulatory T-cells were decreased, changes in TSEN54 expression also failed to affect survival (Figure 12G). This result implied that TSEN54 expression affected patient survival in relation to the degree of infiltration of CD4+ memory T-cells and regulatory T-cells.

### Relationship between TSEN54 and m6A modification in HCC

**Figure 13:**
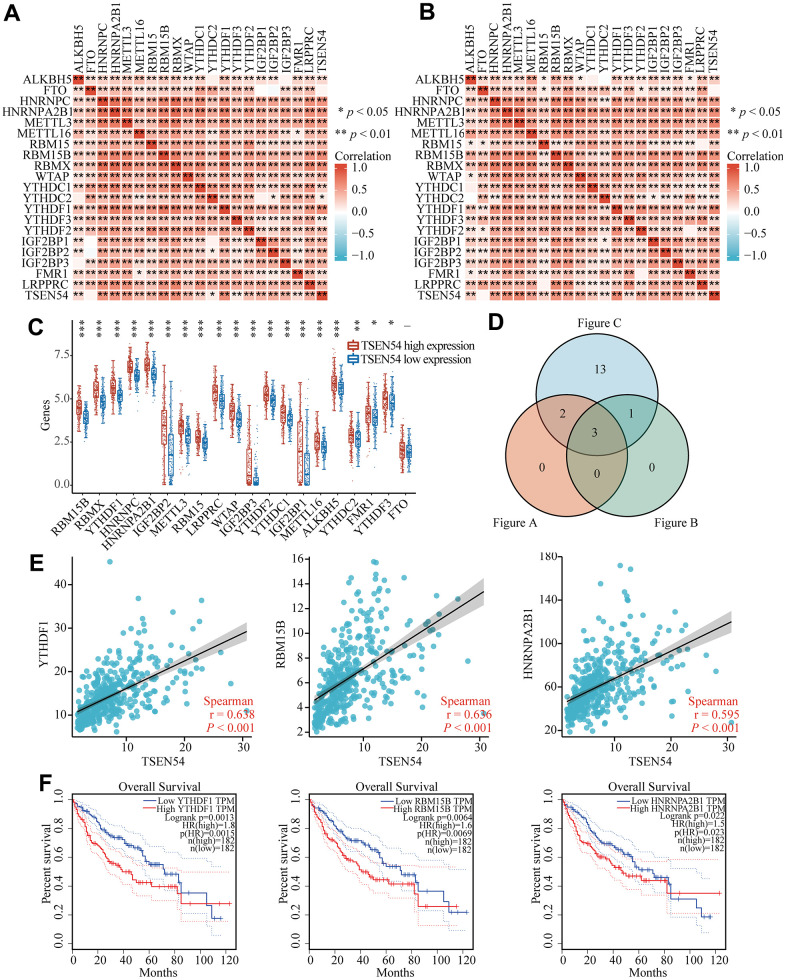
**TSEN54 may be relevant to m6A modification in HCC.** Heatmaps illustrate the association between TSEN54 and regulators relevant to m6A by (**A**) TCGA and (**B**) ICGC databases in HCC. (**C**) The linkage between high and low expression of TSEN54 and the m6A-related regulators in HCC. (**D**) Regulators with expression correlation greater than 0.55 and exhibiting meaningful differential expression are shown by the Venn plot. (**E**) The correlation scatter plots between TSEN54 and 3 regulators in the cross set, including YTHDF1, RBM15B and HNRNPA2B1. (**F**) Survival curves of YTHDF1, RBM15B and HNRNPA2B1 in HCC.

Previous studies have implicated aberrant regulation of m6A modification is associated with a number of human malignancies including HCC, which prompted us to explore its relation to TSEN54 in HCC [41]. In a preliminary step, when we researched the correlation between TSEN54 and the expression of 20 m6A-related regulators through heatmaps on the basis of TCGA and ICGC databases in HCC, we detected that among them, 19 regulators revealed a significantly positive correlation with TSEN54 expression in each of TCGA and ICGC databases (*P*<0.05) (Figure 13A, 13B). In the meantime, dividing the samples into two groups of high and low TSEN54 expression, we studied the associations of these m6A-related regulators with the differential expression of TSEN54, and it was interesting to note that these same 19 regulators had shown remarkable results from Figure 13C. Next, from the Venn plot, we could find three m6A-related regulators whose expression correlation with TSEN54 was greater than 0.55 in ICGC and TCGA databases and presented meaningful differential expression, which were YTHDF1, RBM15B, HNRNPA2B1 (Figure 13D). Immediately after, we plotted the scatter plots of the correlation between these three regulators and TSEN54 (Figure 13E). As a final step, through plotting survival curves on the GEPIA website, we observed that high expression of YTHDF1, RBM15B, and HNRNPA2B1 could lead to low survival of HCC suffers, and it could signal that they may have association with poor prognosis of HCC patients (Figure 13F). Taken together, TSEN54 may have a strong relationship with m6A modification in HCC, and it may play a possible influential part in the progression of HCC patients.

### Network, structure, pathway, and drug sensitivity analysis of TSEN54 and its interacting genes

**Figure 14:**
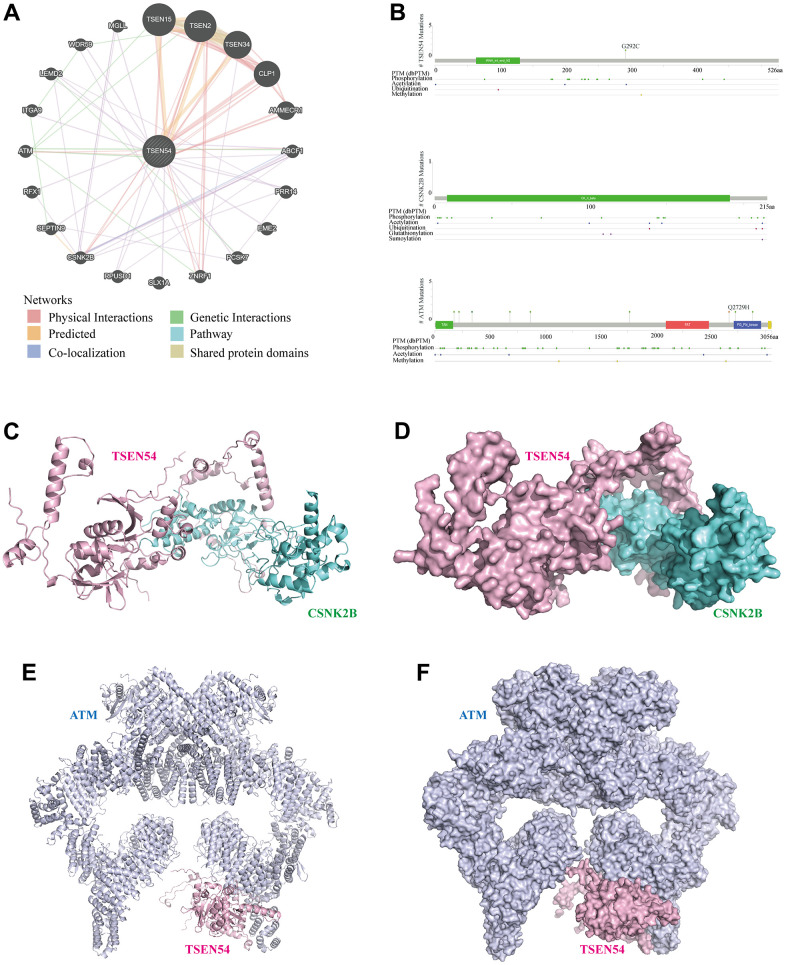
**Analysis of the interaction network and docking structures of TSEN54.** (**A**) Interaction network of TSEN54 with other genes (GeneMANIA). The different colored lines represent different linkage information. (**B**) Protein secondary structures of TSEN54, CSNK2B and ATM. (**C**–**F**) Cartoon view and Surface view of the overall conformation of TSEN54 combined with CSNK2B or ATM. TSEN54 is colored in light pink, CSNK2B is colored in aquamarine, and ATM is colored in blue-white.

**Figure 15:**
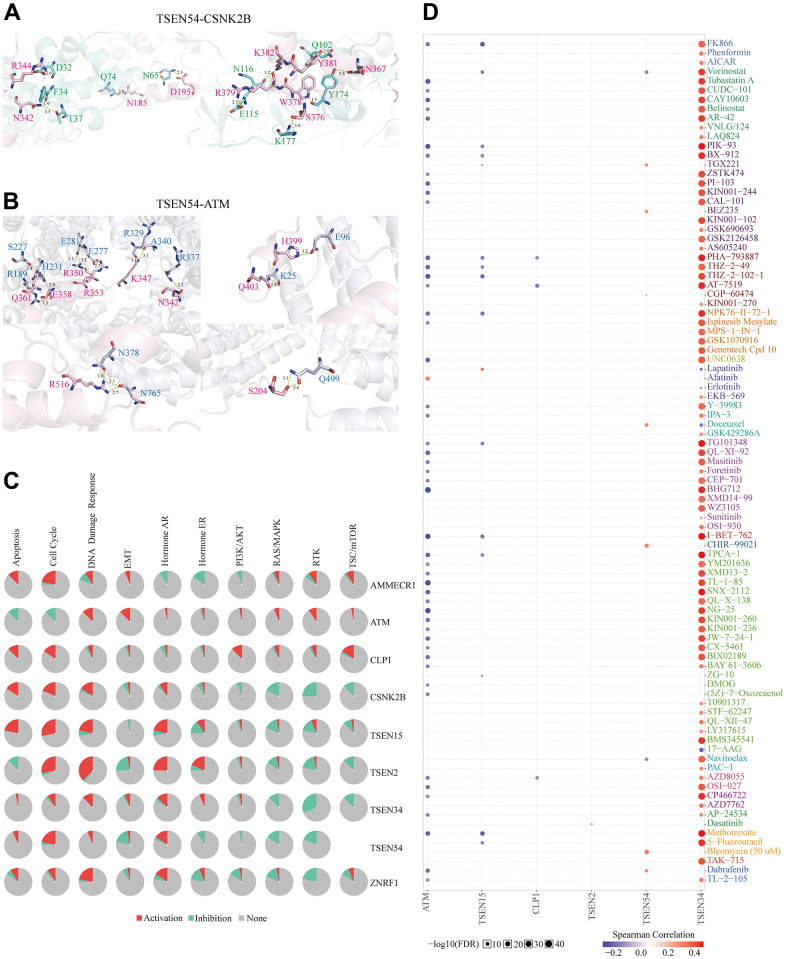
**Analysis of the docking structures, pathways, and drug sensitivity of TSEN54 and its interacting genes.** Detailed interaction structures between (**A**) TSEN54 and CSNK2B and (**B**) TSEN54 and ATM. The key residues of TSEN54 are shown in light pink, CSNK2B in aquamarine, and ATM in blue-white. The hydrogen bonds are shown as yellow dashed lines and the distance between two atoms is marked. (**C**) Cancer-related pathways of TSEN54 and its interacting genes are presented in the form of a global percentage. (**D**) Drug susceptibility analysis of TSEN54 and its interacting genes based on the GDSC database is presented as a bubble chart.

Proteins have specific spatial conformations that can be used to achieve biological functions through interactions. Therefore, we first constructed a network of gene interactions for TSEN54 using the GeneMANIA online website. The eight genes connected to TSEN54 by pink straight lines mean that there exist protein-protein interactions for their coding products (Figure 14A). Surprisingly, we noticed the existence of protein-level physical interactions between TSEN54 and the renowned CSNK2B and ATM. Thus, we further obtained the secondary structures of the proteins encoded by these three genes from the cBioPortal online portal and analyzed their structural domains and protein modification sites. The results showed that TSEN54 has a tRNA_int_end_N2 structural domain with several modification sites, including the phosphorylation site, acetylation site, ubiquitination site, and methylation site. CSNK2B has a CK_II_beta structural domain with a phosphorylation site, acetylation site, ubiquitination site, glutathionylation site, and sumoylation site. ATM has TAN, FAT, PI3_PI4_kinase, with phosphorylation, acetylation, and methylation sites (Figure 14B). Then, we downloaded the spatial structures of CSNK2B, ATM from the PDB database and predicted the TSEN54 spatial structure using AlphaFold Database, and finally predicted their protein-protein interaction docking models using HDOCK Server (Figure 14C–14F). Hydrogen bonds hold a vital position in the binding of protein molecules. In the TSEN54-CSNK2B docking model, we found a total of 11 hydrogen bonds (Figure 15A), and in the TSEN54-ATM docking model, we found a total of 17 hydrogen bonds (Figure 15B).

Furthermore, as we learned from the” pathway activity” module in GSCALite, both TSEN54 and its physical interactions genes could activate notable pathways linked to cancers such as apoptosis, cell cycle, DNA damage response, while inhibiting RAS/MAPK, RTK, and other pathways (Figure 15B). Finally, we performed a gene set resistance analysis of TSEN54 and its physical interactions genes through small molecules collected by the GDSC database. The Spearman correlation gave an indication of genetic expression relating to drug tolerance, where red represented a positive correlation, signifying that cells with high expression of a gene have resistance to a specific drug and blue on the contrary. Thus, we could understand from Figure 15C that cells with high expression of TSEN54 had resistance to 7 drugs such as Bleomycin, and CHIR-99021, while it was sensitive to Vorinostat and Navitoclax. In short, it was possible that these findings may lead to a new way of treating patients with HCC accompanied by high TSEN54 expression.

## DISCUSSION

HCC represents the most prevalent kind of primary liver carcinoma. The complex etiology as well as heightened heterogeneity that characterizes HCC makes early diagnosis and prognosis prediction challenging [42]. Although AFP is the most commonly used biomarker in HCC, its reliability for screening and diagnosis remains suboptimal, as elevated APF level is also seen in gastrointestinal disease or benign liver disease [1]. Therefore, there is an urgent need to discover additional underlying biomarkers aptly guiding both diagnosis and treatment for suffers with HCC. Our research investigated the expression of TSEN54 in HCC using bioinformatics combined with experimental validation, explored the potential modification or regulation mechanisms of TSEN54 at the expression level, predicted HCC patients’ outcome, and further analyzed the biological functions of TSEN54 relevant to HCC development.

Combining the LIHC dataset with clinical samples, we found that TSEN54 expression was generally increased in HCC tissue compared to normal tissue. Subsequently, we found its expression was related to the clinicopathological characteristics and survival of the HCC patients. Notably, the differential expression of TSEN54 showed meaningful result in stage 1 versus normal and grade 1 versus normal, suggesting that the hyper-expression of TSEN54 is related to the early development of HCC which is likely to be an early biomarker for predicting HCC. In addition, AFP as a screening tool for HCC has heterogeneity in AFP expression across geographic regions, and it is suggested that such heterogeneous results are more appropriate in eastern populations and have poor results in western populations [43]. Our results are similar. TSEN54 expression was distinctly higher in the Caucasian, African-American and Asian patient subgroups compared to the normal subgroup, and distinctly higher in the Asian subgroup compared to the Caucasian subgroup, showing the great potential of its expression level in ethnographic differences. TP53, as an important oncogene, whose mutation encodes a protein that loses its normal regulatory transcriptional function and can affect the progression of diverse carcinomas. For example, elevating TPX2 expression facilitates the development of breast cancer, and elevating the expression of H2AFZ promotes the development of hepatocellular carcinoma [44, 45]. Our results showed the TSEN54 expression was clearly upper in the TP53-Mutant group versus the TP53-NonMutant group, suggesting TP53 mutations possibly upregulate the expression level of TSEN54 and thus be involved in the development of HCC.

Methylation of the promoter CpG island hampers the binding of the transcription factor complex to the promoter, hence inhibiting the gene expression [46]. Altogether, our outcomes are in line with this logic that there is a relation between the hyper-expression and hypomethylation of TSEN54 in HCC. From the perspective of methylation, it could partially explain the early expression differences hinted at above, such as stage 1 vs. normal. Whereas discrepancies in TSEN54 promoter methylation level between grade 1 and normal do not appear, pending further exploration of other mechanisms. Noticeably, the TSEN54 promoter methylation level in the TP53-Mutant group in patients was lower than those in the TP53-NonMutant group, which coincidentally corresponded to the higher expression level of TSEN54 in the TP53-Mutant group. This identification offered a fresh idea for TP53 mutations influencing TSEN54 expression.

Multiple approaches point to a poorer prognosis for HCC patients with high TSEN54 expression. High TSEN54 expression also independently predicts poor prognosis in HCC patients. Interestingly, survival curves remained significant in the two-factor subgroup with differential expression of TSEN54 and different HCC classifications, and in the two-factor subgroup with differential expression of TSEN54 and different HCC patient ethnicities, reflecting the strong role of TSEN54 in predicting prognosis in HCC patients. However, comparisons between specific subgroups need to be further investigated.

To deeply elucidate the molecular functions and potential mechanisms of TSEN54 affecting the development of HCC, we performed pathway enrichment analysis. We found that TSEN54, as one of the components listed in the tRNA splicing endonuclease complex, besides participating in RNA splicing and mRNA processing, is also involved in chromatin assembly or disassembly, chromosome segregation, DNA replication, organelle fission, cell cycle phase transition and other biological processes to modulate HCC cells’ cell cycle process, thus participate in the proliferation of hepatocellular carcinoma cells and lead to hepatocarcinogenesis. Meanwhile, overexpression of AZI1, one of the core genes co-expressed with TSEN54, promotes HCC cell growth and migration by activating the PI3K/AKT signaling pathway [47]. Another core gene, CCDC137, is also overexpressed in diverse tumors, including HCC [48]. It has also been found that CCDC137 depletion leads to G2/M cell cycle arrest [49]. In conclusion, TSEN54 is presumably involved in cell cycle regulation and directly affects hepatocellular carcinogenesis.

Although TSEN54 is associated with multiple DNA repair responses, an inappropriate and untimely repair can still lead to genomic alterations that provide the molecular basis for cancer development. Notably, homologous recombination and Fanconi anemia pathway are included as pathways associated with TSEN54, and the primary mechanism of Fanconi anemia etiopathogenesis coincides with defects in double-stranded DNA homologous recombination. Diverse FA proteins sustain genomic stabilization by DNA interstrand crosslinking (ICL) repair [50]. It has been reported that FANCD2 is overexpressed to participate in the proliferation and invasion of HCC cells and leads to HCC progression [51]. Enhanced expression of FA / BRCA pathway genes has also been reported to be an important mechanism of chemoresistance in HCC [52]. Whether TSEN54 is associated with gene repair function and Fanconi anemia pathway leading to HCC cell progression still awaits deeper investigation through molecular experiments.

Moreover, metabolic reprogramming is one of the important features of cancer cells and is no exception in HCC cells [53, 54]. In glucose metabolism, the Warburg effect suggests that glucose is rarely completely oxidized in cancer cells even in an aerobic environment, but tends to produce ATP and lactate via glycolysis. Although such an alteration would be inefficient for meeting ATP requirements, an increase in intermediates in the glycolytic pathway would provide additional benefits for tumor growth. This is echoed by the downregulation of the TCA cycle pathway in the results, implying more accumulation of intermediates and less energy production by oxidative phosphorylation. The downregulation of fatty acid metabolism and fatty acid degradation pathway was also observed in our results, suggesting more accumulation of fatty acids. It has been suggested that during the development of HCC, fatty acids serve as signal precursors to regulate metabolism and as energy sources to support rapid cell proliferation, cell survival, invasion, and angiogenesis [55]. In short, TSEN54 may be involved in metabolic reprogramming, leading to enhanced cellular malignant behavior and laying the material and energy basis for the development of HCC.

HCC is inflammation-associated cancer in which prolonged and recurrent inflammation leads to immune cell infiltration with alterations in the composition of the liver microenvironment, and the specific microenvironment will contribute to the malignant transformation of hepatocytes and drive cancer development [56]. Our study showed that TSEN54 is commonly expressed in various immune cells. TSEN54 expression showed a positive correlation with immune cell infiltration like B cells, CD4+ T cells, macrophages, and dendritic cells, and positively correlated with markers of multiple immune cells. In the second paragraph, we mentioned that TP53 mutation has been correlated with high expression level of TSEN54. And the results of the immune section indicated that TSEN54 expression was positively related to macrophage and B-cell infiltration mainly. Interestingly, the TP53 mutation group in the results happened to have a higher level of B-cell and macrophage infiltration. This finding provides a novel idea for TP53 and TSEN54 to regulate the immune microenvironment of HCC and thus participate in tumor progression.

Although TSEN54 expression was related to multiple immune cells infiltration and expression of chemokines such as CCL15, CCL26, and CCL28, it was CD4+ memory T-cells and regulatory T-cells which actually affected the survival expectations of patients with differential TSEN54 expression. Results showed that enriched CD4 + memory T-cells resisted the poor prognosis associated with high TSEN54 expression, while decreased Regulatory T-cells also resisted the poor outcome associated with high TSEN54 expression. CD4+ memory T-cells can proliferate and differentiate faster after receiving antigen stimulation for a second time, and the differentiated subpopulation exerts anti-tumor effects by secreting cytokines or activating CD8+ T lymphocytes [57, 58]. In contrast, Regulatory T-cells help HCC cells to escape from immune surveillance by suppressing the proliferation of effector T cells and reducing the antitumor immune response, which in turn enhances the invasive and metastatic ability of HCC [59]. Similar to our results, it has likewise been suggested that rescuing CD4+ T lymphocytes from apoptosis can halt the development of HCC and that removing or reducing Treg can improve the efficacy of immunotherapy for HCC [60, 61]. This finding provides a promising direction for treating patients with differential TSEN54 expression and improving survival expectations.

Immune checkpoints, especially PD-1 and CTLA4, are involved in tumor immune escape by generating inhibitory signals that suppress the activation or proliferation of some immune cells [62]. In addition, CD276 (B7-H3), a newly identified immunomodulatory protein, also has a strong immunosuppressive effect and also promotes vasculogenic mimicry formation in the hepatocellular carcinoma microenvironment [63, 64]. Our study detected a positive association between the TSEN54 expression level and CD276, CTLA4, PDCD1, etc. It is speculated that the high expression of TSEN54 is probably engaged in the immune escape process of HCC cells, which, on the other hand, provides more possibilities for the application of immune checkpoint inhibitors (ICI).

N6-methyladenosine (m6A) as the richest mRNA modification in eukaryotes is participated in almost every step of RNA metabolism, regulating cell turnover, differentiation, and apoptotic processes by regulating gene expression [65]. Increasing evidence suggests that m6A modification and aberrant expression of m6A enzymes perform essentially in the progression of HCC [66]. Therefore, we surveyed the correlation of TSEN54 with m6A-related regulators and targeted three intersecting regulators (YTHDF1, RBM15B, HNRNPA2B1) through both ICGC and TCGA databases. Coincidentally, all three regulators were reported over expressed in hepatocellular carcinoma and involved in the development of hepatocellular carcinoma [67–69]. For example, RBM15B facilitates the proliferation and invasion of HCC cells through the YY1-RBM15B-TRAM2 regulatory axis [68]. Overall, there exists a robust correlation between TSEN54 and m6A-related regulators, particularly the three intersecting regulators mentioned above, which implies that TSEN54 may influence the m6A modification process and thus the progression of hepatocellular carcinoma.

The interaction network suggests that TSEN54 interacts with CSNK2B, ATM at the physical level. Casein kinase 2 beta (CSNK2B) codifies the beta sub-unit of casein kinase II (CK2). CK2 can engage in modulating various biological processes such as cell proliferation, differentiation, and survival by phosphorylating target proteins. Increasing researches showed the important utility of CSNK2B in diversified carcinomas, e.g., hepatocellular carcinoma, gastric cancer, breast cancer, and colorectal cancer [70–73]. In addition, ATM, as a major player in the DNA damage response, can phosphorylate downstream targets upon activation, thereby regulating progression with regard to DNA damage repair, apoptosis, and cell cycle arrest [74]. It has been suggested that ATM expression is lower in hepatocellular carcinoma and leads to poor patient outcomes [75]. In addition, the results of the interaction network also indicated a genetic interaction between TSEN54 and ATM, whilst the secondary structure revealed the presence of phosphorylation modification sites for TSEN54. This finding strengthens the link between the two and enriches the possible mechanisms by which TSEN54 regulates the progression of hepatocellular carcinoma. To better reveal these two pairs of interacting proteins, we also predicted the possible binding sites by molecular docking, which will provide a basis for future experimental studies. Finally, we further identified that HCC cells with highly expressed TSEN54 were sensitive to two small molecule drugs (Navitoclax and Vorinostat), providing new ideas for the precise therapeutic of hepatocellular carcinoma.

In summary, TSEN54 is hyper expressed and hypomethylated in hepatocellular carcinoma and is significantly correlated with clinicopathological staging, grading, and poor prognosis. TSEN54 is linked to the cell cycle, DNA damage repair, metabolic reprogramming and immune cell infiltration. Also, TSEN54 is closely associated with related regulators involved in m6A modification. TSEN54 exhibits physical interactions with CSNK2B and ATM. HCC cells which are highly expressing TSEN54 are sensitive to selected drugs. Our study suggests that TSEN54 might serve as a diagnostic and immune-related therapeutic target for hepatocellular carcinoma and provides a new direction for precision drug administration.

## Supplementary Material

Supplementary Figures

Supplementary Tables

## References

[1] Galle PR, Foerster F, Kudo M, Chan SL, Llovet JM, Qin S, Schelman WR, Chintharlapalli S, Abada PB, Sherman M, Zhu AX. Biology and significance of alpha-fetoprotein in hepatocellular carcinoma. Liver Int. 2019; 39:2214–29. 10.1111/liv.14223 31436873

[2] Yu R, Tan Z, Xiang X, Dan Y, Deng G. Effectiveness of PIVKA-II in the detection of hepatocellular carcinoma based on real-world clinical data. BMC Cancer. 2017; 17:608. 10.1186/s12885-017-3609-6 28863782 PMC5580438

[3] Luo P, Wu S, Yu Y, Ming X, Li S, Zuo X, Tu J. Current Status and Perspective Biomarkers in AFP Negative HCC: Towards Screening for and Diagnosing Hepatocellular Carcinoma at an Earlier Stage. Pathol Oncol Res. 2020; 26:599–603. 10.1007/s12253-019-00585-5 30661224

[4] Thein HH, Qiao Y, Zaheen A, Jembere N, Sapisochin G, Chan KK, Yoshida EM, Earle CC. Cost-effectiveness analysis of treatment with non-curative or palliative intent for hepatocellular carcinoma in the real-world setting. PLoS One. 2017; 12:e0185198. 10.1371/journal.pone.0185198 29016627 PMC5634563

[5] Mehta N, Dodge JL, Grab JD, Yao FY. National Experience on Down-Staging of Hepatocellular Carcinoma Before Liver Transplant: Influence of Tumor Burden, Alpha-Fetoprotein, and Wait Time. Hepatology. 2020; 71:943–54. 10.1002/hep.30879 31344273 PMC8722406

[6] Zhu AX, Kang YK, Yen CJ, Finn RS, Galle PR, Llovet JM, Assenat E, Brandi G, Pracht M, Lim HY, Rau KM, Motomura K, Ohno I, et al, and REACH-2 study investigators. Ramucirumab after sorafenib in patients with advanced hepatocellular carcinoma and increased α-fetoprotein concentrations (REACH-2): a randomised, double-blind, placebo-controlled, phase 3 trial. Lancet Oncol. 2019; 20:282–96. 10.1016/S1470-2045(18)30937-9 30665869

[7] Wang J, Ha J, Lopez A, Bhuket T, Liu B, Wong RJ. Medicaid and Uninsured Hepatocellular Carcinoma Patients Have More Advanced Tumor Stage and Are Less Likely to Receive Treatment. J Clin Gastroenterol. 2018. 10.1097/MCG.0000000000000859 28723861

[8] Kulik L, Heimbach JK, Zaiem F, Almasri J, Prokop LJ, Wang Z, Murad MH, Mohammed K. Therapies for patients with hepatocellular carcinoma awaiting liver transplantation: A systematic review and meta-analysis. Hepatology. 2018; 67:381–400. 10.1002/hep.29485 28859222

[9] Lee JR, Kim YH, Park SJ, Choe SH, Cho HM, Lee SR, Kim SU, Kim JS, Sim BW, Song BS, Jeong KJ, Lee Y, Jin YB, et al. Identification of Alternative Variants and Insertion of the Novel Polymorphic *AluYl17* in *TSEN54* Gene during Primate Evolution. Int J Genomics. 2016; 2016:1679574. 10.1155/2016/1679574 28083540 PMC5204098

[10] Abelson J, Trotta CR, Li H. tRNA splicing. J Biol Chem. 1998; 273:12685–8. 10.1074/jbc.273.21.12685 9582290

[11] Budde BS, Namavar Y, Barth PG, Poll-The BT, Nürnberg G, Becker C, van Ruissen F, Weterman MA, Fluiter K, te Beek ET, Aronica E, van der Knaap MS, Höhne W, et al. tRNA splicing endonuclease mutations cause pontocerebellar hypoplasia. Nat Genet. 2008; 40:1113–8. 10.1038/ng.204 18711368

[12] Cassandrini D, Biancheri R, Tessa A, Di Rocco M, Di Capua M, Bruno C, Denora PS, Sartori S, Rossi A, Nozza P, Emma F, Mezzano P, Politi MR, et al. Pontocerebellar hypoplasia: clinical, pathologic, and genetic studies. Neurology. 2010; 75:1459–64. 10.1212/WNL.0b013e3181f88173 20956791

[13] Lou S, Meng F, Yin X, Zhang Y, Han B, Xue Y. Comprehensive Characterization of RNA Processing Factors in Gastric Cancer Identifies a Prognostic Signature for Predicting Clinical Outcomes and Therapeutic Responses. Front Immunol. 2021; 12:719628. 10.3389/fimmu.2021.719628 34413861 PMC8369824

[14] Lai D, Weng S, Wang C, Qi L, Yu C, Fu L, Chen W. Small antisense RNA to cyclin D1 generated by pre-tRNA splicing inhibits growth of human hepatoma cells. FEBS Lett. 2004; 576:481–6. 10.1016/j.febslet.2004.09.040 15498584

[15] Tomczak K, Czerwińska P, Wiznerowicz M. The Cancer Genome Atlas (TCGA): an immeasurable source of knowledge. Contemp Oncol (Pozn). 2015; 19:A68–77. 10.5114/wo.2014.47136 25691825 PMC4322527

[16] Li T, Fan J, Wang B, Traugh N, Chen Q, Liu JS, Li B, Liu XS. TIMER: A Web Server for Comprehensive Analysis of Tumor-Infiltrating Immune Cells. Cancer Res. 2017; 77:e108–10. 10.1158/0008-5472.CAN-17-0307 29092952 PMC6042652

[17] Lian Q, Wang S, Zhang G, Wang D, Luo G, Tang J, Chen L, Gu J. HCCDB: A Database of Hepatocellular Carcinoma Expression Atlas. Genomics Proteomics Bioinformatics. 2018; 16:269–75. 10.1016/j.gpb.2018.07.003 30266410 PMC6205074

[18] Tang Z, Li C, Kang B, Gao G, Li C, Zhang Z. GEPIA: a web server for cancer and normal gene expression profiling and interactive analyses. Nucleic Acids Res. 2017; 45:W98–102. 10.1093/nar/gkx247 28407145 PMC5570223

[19] Thul PJ, Lindskog C. The human protein atlas: A spatial map of the human proteome. Protein Sci. 2018; 27:233–44. 10.1002/pro.3307 28940711 PMC5734309

[20] Chandrashekar DS, Bashel B, Balasubramanya SA, Creighton CJ, Ponce-Rodriguez I, Chakravarthi BV, Varambally S. UALCAN: A Portal for Facilitating Tumor Subgroup Gene Expression and Survival Analyses. Neoplasia. 2017; 19:649–58. 10.1016/j.neo.2017.05.002 28732212 PMC5516091

[21] Gao J, Aksoy BA, Dogrusoz U, Dresdner G, Gross B, Sumer SO, Sun Y, Jacobsen A, Sinha R, Larsson E, Cerami E, Sander C, Schultz N. Integrative analysis of complex cancer genomics and clinical profiles using the cBioPortal. Sci Signal. 2013; 6:pl1. 10.1126/scisignal.2004088 23550210 PMC4160307

[22] Koch A, Jeschke J, Van Criekinge W, van Engeland M, De Meyer T. MEXPRESS update 2019. Nucleic Acids Res. 2019; 47:W561–5. 10.1093/nar/gkz445 31114869 PMC6602516

[23] Li Y, Ge D, Lu C. The SMART App: an interactive web application for comprehensive DNA methylation analysis and visualization. Epigenetics Chromatin. 2019; 12:71. 10.1186/s13072-019-0316-3 31805986 PMC6894252

[24] McGeary SE, Lin KS, Shi CY, Pham TM, Bisaria N, Kelley GM, Bartel DP. The biochemical basis of microRNA targeting efficacy. Science. 2019; 366:eaav1741. 10.1126/science.aav1741 31806698 PMC7051167

[25] Kang J, Tang Q, He J, Li L, Yang N, Yu S, Wang M, Zhang Y, Lin J, Cui T, Hu Y, Tan P, Cheng J, et al. RNAInter v4.0: RNA interactome repository with redefined confidence scoring system and improved accessibility. Nucleic Acids Res. 2022; 50:D326–32. 10.1093/nar/gkab997 34718726 PMC8728132

[26] Chang L, Zhou G, Soufan O, Xia J. miRNet 2.0: network-based visual analytics for miRNA functional analysis and systems biology. Nucleic Acids Res. 2020; 48:W244–51. 10.1093/nar/gkaa467 32484539 PMC7319552

[27] Li JH, Liu S, Zhou H, Qu LH, Yang JH. starBase v2.0: decoding miRNA-ceRNA, miRNA-ncRNA and protein-RNA interaction networks from large-scale CLIP-Seq data. Nucleic Acids Res. 2014; 42:D92–7. 10.1093/nar/gkt1248 24297251 PMC3964941

[28] Lánczky A, Győrffy B. Web-Based Survival Analysis Tool Tailored for Medical Research (KMplot): Development and Implementation. J Med Internet Res. 2021; 23:e27633. 10.2196/27633 34309564 PMC8367126

[29] Vasaikar SV, Straub P, Wang J, Zhang B. LinkedOmics: analyzing multi-omics data within and across 32 cancer types. Nucleic Acids Res. 2018; 46:D956–63. 10.1093/nar/gkx1090 29136207 PMC5753188

[30] Subramanian A, Tamayo P, Mootha VK, Mukherjee S, Ebert BL, Gillette MA, Paulovich A, Pomeroy SL, Golub TR, Lander ES, Mesirov JP. Gene set enrichment analysis: a knowledge-based approach for interpreting genome-wide expression profiles. Proc Natl Acad Sci USA. 2005; 102:15545–50. 10.1073/pnas.0506580102 16199517 PMC1239896

[31] Sun D, Wang J, Han Y, Dong X, Ge J, Zheng R, Shi X, Wang B, Li Z, Ren P, Sun L, Yan Y, Zhang P, et al. TISCH: a comprehensive web resource enabling interactive single-cell transcriptome visualization of tumor microenvironment. Nucleic Acids Res. 2021; 49:D1420–30. 10.1093/nar/gkaa1020 33179754 PMC7778907

[32] Ru B, Wong CN, Tong Y, Zhong JY, Zhong SS, Wu WC, Chu KC, Wong CY, Lau CY, Chen I, Chan NW, Zhang J. TISIDB: an integrated repository portal for tumor-immune system interactions. Bioinformatics. 2019; 35:4200–2. 10.1093/bioinformatics/btz210 30903160

[33] Leng X, Liu G, Wang S, Song J, Zhang W, Zhang X, Rong L, Ma Y, Song F. LINC01272 Promotes Migration and Invasion of Gastric Cancer Cells via EMT. Onco Targets Ther. 2020; 13:3401–10. 10.2147/OTT.S242073 32368096 PMC7184168

[34] Franz M, Rodriguez H, Lopes C, Zuberi K, Montojo J, Bader GD, Morris Q. GeneMANIA update 2018. Nucleic Acids Res. 2018; 46:W60–4. 10.1093/nar/gky311 29912392 PMC6030815

[35] Cerami E, Gao J, Dogrusoz U, Gross BE, Sumer SO, Aksoy BA, Jacobsen A, Byrne CJ, Heuer ML, Larsson E, Antipin Y, Reva B, Goldberg AP, et al. The cBio cancer genomics portal: an open platform for exploring multidimensional cancer genomics data. Cancer Discov. 2012; 2:401–4. 10.1158/2159-8290.CD-12-0095 22588877 PMC3956037

[36] Berman HM, Westbrook J, Feng Z, Gilliland G, Bhat TN, Weissig H, Shindyalov IN, Bourne PE. The Protein Data Bank. Nucleic Acids Res. 2000; 28:235–42. 10.1093/nar/28.1.235 10592235 PMC102472

[37] Jumper J, Evans R, Pritzel A, Green T, Figurnov M, Ronneberger O, Tunyasuvunakool K, Bates R, Žídek A, Potapenko A, Bridgland A, Meyer C, Kohl SA, et al. Highly accurate protein structure prediction with AlphaFold. Nature. 2021; 596:583–9. 10.1038/s41586-021-03819-2 34265844 PMC8371605

[38] Remmert M, Biegert A, Hauser A, Söding J. HHblits: lightning-fast iterative protein sequence searching by HMM-HMM alignment. Nat Methods. 2011; 9:173–5. 10.1038/nmeth.1818 22198341

[39] Liu CJ, Hu FF, Xia MX, Han L, Zhang Q, Guo AY. GSCALite: a web server for gene set cancer analysis. Bioinformatics. 2018; 34:3771–2. 10.1093/bioinformatics/bty411 29790900

[40] Klutstein M, Nejman D, Greenfield R, Cedar H. DNA Methylation in Cancer and Aging. Cancer Res. 2016; 76:3446–50. 10.1158/0008-5472.CAN-15-3278 27256564

[41] Liu ZX, Li LM, Sun HL, Liu SM. Link Between m6A Modification and Cancers. Front Bioeng Biotechnol. 2018; 6:89. 10.3389/fbioe.2018.00089 30062093 PMC6055048

[42] Liang JY, Wang DS, Lin HC, Chen XX, Yang H, Zheng Y, Li YH. A Novel Ferroptosis-related Gene Signature for Overall Survival Prediction in Patients with Hepatocellular Carcinoma. Int J Biol Sci. 2020; 16:2430–41. 10.7150/ijbs.45050 32760210 PMC7378635

[43] Piñero F, Dirchwolf M, Pessôa MG. Biomarkers in Hepatocellular Carcinoma: Diagnosis, Prognosis and Treatment Response Assessment. Cells. 2020; 9:1370. 10.3390/cells9061370 32492896 PMC7349517

[44] Matson DR, Denu RA, Zasadil LM, Burkard ME, Weaver BA, Flynn C, Stukenberg PT. High nuclear TPX2 expression correlates with TP53 mutation and poor clinical behavior in a large breast cancer cohort, but is not an independent predictor of chromosomal instability. BMC Cancer. 2021; 21:186. 10.1186/s12885-021-07893-7 33622270 PMC7901195

[45] Dong M, Chen J, Deng Y, Zhang D, Dong L, Sun D. H2AFZ Is a Prognostic Biomarker Correlated to TP53 Mutation and Immune Infiltration in Hepatocellular Carcinoma. Front Oncol. 2021; 11:701736. 10.3389/fonc.2021.701736 34760688 PMC8573175

[46] Zhu H, Wang G, Qian J. Transcription factors as readers and effectors of DNA methylation. Nat Rev Genet. 2016; 17:551–65. 10.1038/nrg.2016.83 27479905 PMC5559737

[47] Liu XH, Yang YF, Fang HY, Wang XH, Zhang MF, Wu DC. CEP131 indicates poor prognosis and promotes cell proliferation and migration in hepatocellular carcinoma. Int J Biochem Cell Biol. 2017; 90:1–8. 10.1016/j.biocel.2017.07.001 28694105

[48] Guo L, Li B, Lu Z, Liang H, Yang H, Chen Y, Zhu S, Zeng M, Wei Y, Liu T, Jiang T, Xuan M, Tang H. CCDC137 Is a Prognostic Biomarker and Correlates With Immunosuppressive Tumor Microenvironment Based on Pan-Cancer Analysis. Front Mol Biosci. 2021; 8:674863. 10.3389/fmolb.2021.674863 34055889 PMC8155610

[49] Zhang F, Bieniasz PD. HIV-1 Vpr induces cell cycle arrest and enhances viral gene expression by depleting CCDC137. Elife. 2020; 9:e55806. 10.7554/eLife.55806 32538781 PMC7295576

[50] Bhandari J, Thada PK, Puckett Y. Fanconi Anemia. 2022. In: StatPearls. Treasure Island (FL): StatPearls Publishing. 2023. 32644559

[51] Komatsu H, Masuda T, Iguchi T, Nambara S, Sato K, Hu Q, Hirata H, Ito S, Eguchi H, Sugimachi K, Eguchi H, Doki Y, Mori M, Mimori K. Clinical Significance of *FANCD2* Gene Expression and its Association with Tumor Progression in Hepatocellular Carcinoma. Anticancer Res. 2017; 37:1083–90. 10.21873/anticanres.11420 28314268

[52] Ferroudj S, Yildiz G, Bouras M, Iscan E, Ekin U, Ozturk M. Role of Fanconi anemia/BRCA pathway genes in hepatocellular carcinoma chemoresistance. Hepatol Res. 2016; 46:1264–74. 10.1111/hepr.12675 26885668

[53] Gyamfi J, Kim J, Choi J. Cancer as a Metabolic Disorder. Int J Mol Sci. 2022; 23:1155. 10.3390/ijms23031155 35163079 PMC8835572

[54] Du D, Liu C, Qin M, Zhang X, Xi T, Yuan S, Hao H, Xiong J. Metabolic dysregulation and emerging therapeutical targets for hepatocellular carcinoma. Acta Pharm Sin B. 2022; 12:558–80. 10.1016/j.apsb.2021.09.019 35256934 PMC8897153

[55] Wang M, Han J, Xing H, Zhang H, Li Z, Liang L, Li C, Dai S, Wu M, Shen F, Yang T. Dysregulated fatty acid metabolism in hepatocellular carcinoma. Hepat Oncol. 2016; 3:241–51. 10.2217/hep-2016-0012 30191046 PMC6095185

[56] Sas Z, Cendrowicz E, Weinhäuser I, Rygiel TP. Tumor Microenvironment of Hepatocellular Carcinoma: Challenges and Opportunities for New Treatment Options. Int J Mol Sci. 2022; 23:3778. 10.3390/ijms23073778 35409139 PMC8998420

[57] Salek-Ardakani S, Croft M. Regulation of CD4 T cell memory by OX40 (CD134). Vaccine. 2006; 24:872–83. 10.1016/j.vaccine.2005.07.108 16176850

[58] Zheng X, Jin W, Wang S, Ding H. Progression on the Roles and Mechanisms of Tumor-Infiltrating T Lymphocytes in Patients With Hepatocellular Carcinoma. Front Immunol. 2021; 12:729705. 10.3389/fimmu.2021.729705 34566989 PMC8462294

[59] Bozward AG, Warricker F, Oo YH, Khakoo SI. Natural Killer Cells and Regulatory T Cells Cross Talk in Hepatocellular Carcinoma: Exploring Therapeutic Options for the Next Decade. Front Immunol. 2021; 12:643310. 10.3389/fimmu.2021.643310 33995362 PMC8120158

[60] Brown ZJ, Fu Q, Ma C, Kruhlak M, Zhang H, Luo J, Heinrich B, Yu SJ, Zhang Q, Wilson A, Shi ZD, Swenson R, Greten TF. Carnitine palmitoyltransferase gene upregulation by linoleic acid induces CD4^+^ T cell apoptosis promoting HCC development. Cell Death Dis. 2018; 9:620. 10.1038/s41419-018-0687-6 29795111 PMC5966464

[61] Yu S, Wang Y, Hou J, Li W, Wang X, Xiang L, Tan D, Wang W, Jiang L, Claret FX, Jiao M, Guo H. Tumor-infiltrating immune cells in hepatocellular carcinoma: Tregs is correlated with poor overall survival. PLoS One. 2020; 15:e0231003. 10.1371/journal.pone.0231003 32240238 PMC7117689

[62] Xing R, Gao J, Cui Q, Wang Q. Strategies to Improve the Antitumor Effect of Immunotherapy for Hepatocellular Carcinoma. Front Immunol. 2021; 12:783236. 10.3389/fimmu.2021.783236 34899747 PMC8660685

[63] Zhou WT, Jin WL. B7-H3/CD276: An Emerging Cancer Immunotherapy. Front Immunol. 2021; 12:701006. 10.3389/fimmu.2021.701006 34349762 PMC8326801

[64] Cheng R, Wang B, Cai XR, Chen ZS, Du Q, Zhou LY, Ye JM, Chen YL. CD276 Promotes Vasculogenic Mimicry Formation in Hepatocellular Carcinoma via the PI3K/AKT/MMPs Pathway. Onco Targets Ther. 2020; 13:11485–98. 10.2147/OTT.S271891 33204103 PMC7667184

[65] Chen J, Zhang Q, Liu T, Tang H. Roles of M^6^A Regulators in Hepatocellular Carcinoma: Promotion or Suppression. Curr Gene Ther. 2022; 22:40–50. 10.2174/1566523221666211126105940 34825870

[66] Wang YF, Ge CM, Yin HZ, Dai ZH, Dong JP, Ji M, Yang F. Dysregulated N6-methyladenosine (m^6^A) processing in hepatocellular carcinoma. Ann Hepatol. 2021; 25:100538. 10.1016/j.aohep.2021.100538 34555511

[67] Luo X, Cao M, Gao F, He X. YTHDF1 promotes hepatocellular carcinoma progression via activating PI3K/AKT/mTOR signaling pathway and inducing epithelial-mesenchymal transition. Exp Hematol Oncol. 2021; 10:35. 10.1186/s40164-021-00227-0 34088349 PMC8176587

[68] Tan C, Xia P, Zhang H, Xu K, Liu P, Guo D, Liu Z. YY1-Targeted RBM15B Promotes Hepatocellular Carcinoma Cell Proliferation and Sorafenib Resistance by Promoting TRAM2 Expression in an m6A-Dependent Manner. Front Oncol. 2022; 12:873020. 10.3389/fonc.2022.873020 35494016 PMC9046568

[69] Zhou B, Lu D, Wang A, Cui J, Zhang L, Li J, Fan L, Wei W, Liu J, Sun G. Endoplasmic reticulum stress promotes sorafenib resistance via miR-188-5p/hnRNPA2B1-mediated upregulation of PKM2 in hepatocellular carcinoma. Mol Ther Nucleic Acids. 2021; 26:1051–65. 10.1016/j.omtn.2021.09.014 34786210 PMC8569435

[70] Xiao Y, Huang S, Qiu F, Ding X, Sun Y, Wei C, Hu X, Wei K, Long S, Xie L, Xun Y, Chen W, Zhang Z, et al. Tumor necrosis factor α-induced protein 1 as a novel tumor suppressor through selective downregulation of CSNK2B blocks nuclear factor-κB activation in hepatocellular carcinoma. EBioMedicine. 2020; 51:102603. 10.1016/j.ebiom.2019.102603 31901862 PMC6950786

[71] Yu S, Li L, Cai H, He B, Gao Y, Li Y. Overexpression of NELFE contributes to gastric cancer progression via Wnt/β-catenin signaling-mediated activation of CSNK2B expression. J Exp Clin Cancer Res. 2021; 40:54. 10.1186/s13046-021-01848-3 33526068 PMC7851912

[72] Wang W, Wang X, Li C, Chen T, Zhang N, Liang Y, Li Y, Zhang H, Liu Y, Song X, Zhao W, Chen B, Wang L, Yang Q. Huaier Suppresses Breast Cancer Progression via linc00339/miR-4656/CSNK2B Signaling Pathway. Front Oncol. 2019; 9:1195. 10.3389/fonc.2019.01195 31781497 PMC6857111

[73] Yu S, Hu Q, Fan K, Yang C, Gao Y. CSNK2B contributes to colorectal cancer cell proliferation by activating the mTOR signaling. J Cell Commun Signal. 2021; 15:383–92. 10.1007/s12079-021-00619-1 33928514 PMC8222461

[74] Phan LM, Rezaeian AH. ATM: Main Features, Signaling Pathways, and Its Diverse Roles in DNA Damage Response, Tumor Suppression, and Cancer Development. Genes (Basel). 2021; 12:845. 10.3390/genes12060845 34070860 PMC8228802

[75] Yan X, Wu T, Tang M, Chen D, Huang M, Zhou S, Zhang H, Yang X, Li G. Methylation of the ataxia telangiectasia mutated gene (ATM) promoter as a radiotherapy outcome biomarker in patients with hepatocellular carcinoma. Medicine (Baltimore). 2020; 99:e18823. 10.1097/MD.0000000000018823 31977876 PMC7004781

