# Supplementary material for: Comprehensive analysis reveals TSEN54 as a robust prognosis biomarker and promising immune-related therapeutic target for hepatocellular carcinoma
Source: Aging (Albany NY). 2023 Apr 8;15(7):2734–71. doi: 10.18632/aging.204645 (PMC10120902; doi:10.18632/aging.204645)
Supplement: Supplementary Figures [file aging-15-204645-s001.pdf]

SUPPLEMENTARY FIGURES

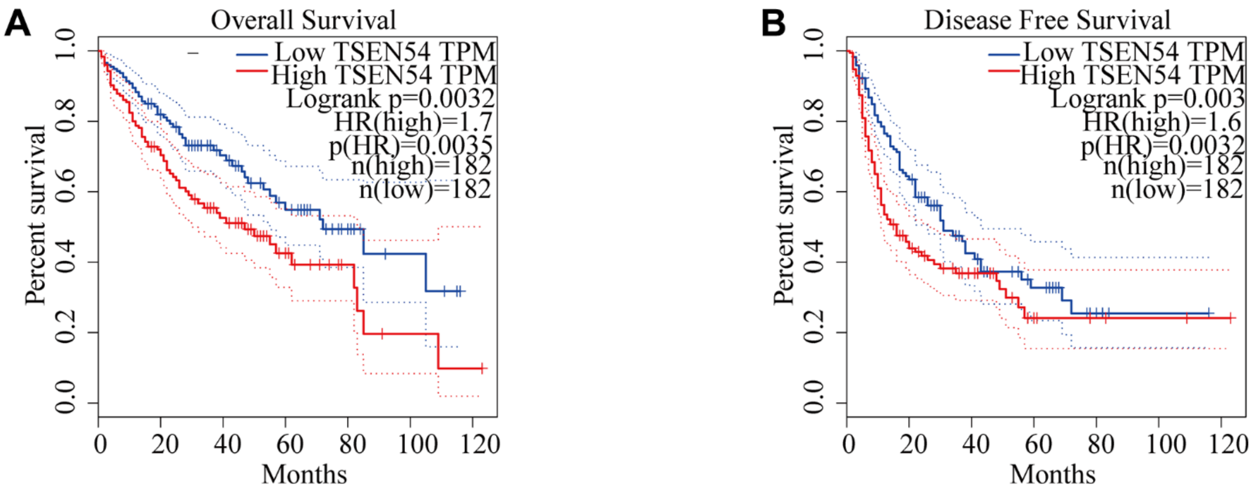

Supplementary Figure 1. Survival curves using the GEPIA website are shown for (**A**) OS and (**B**) DFS.

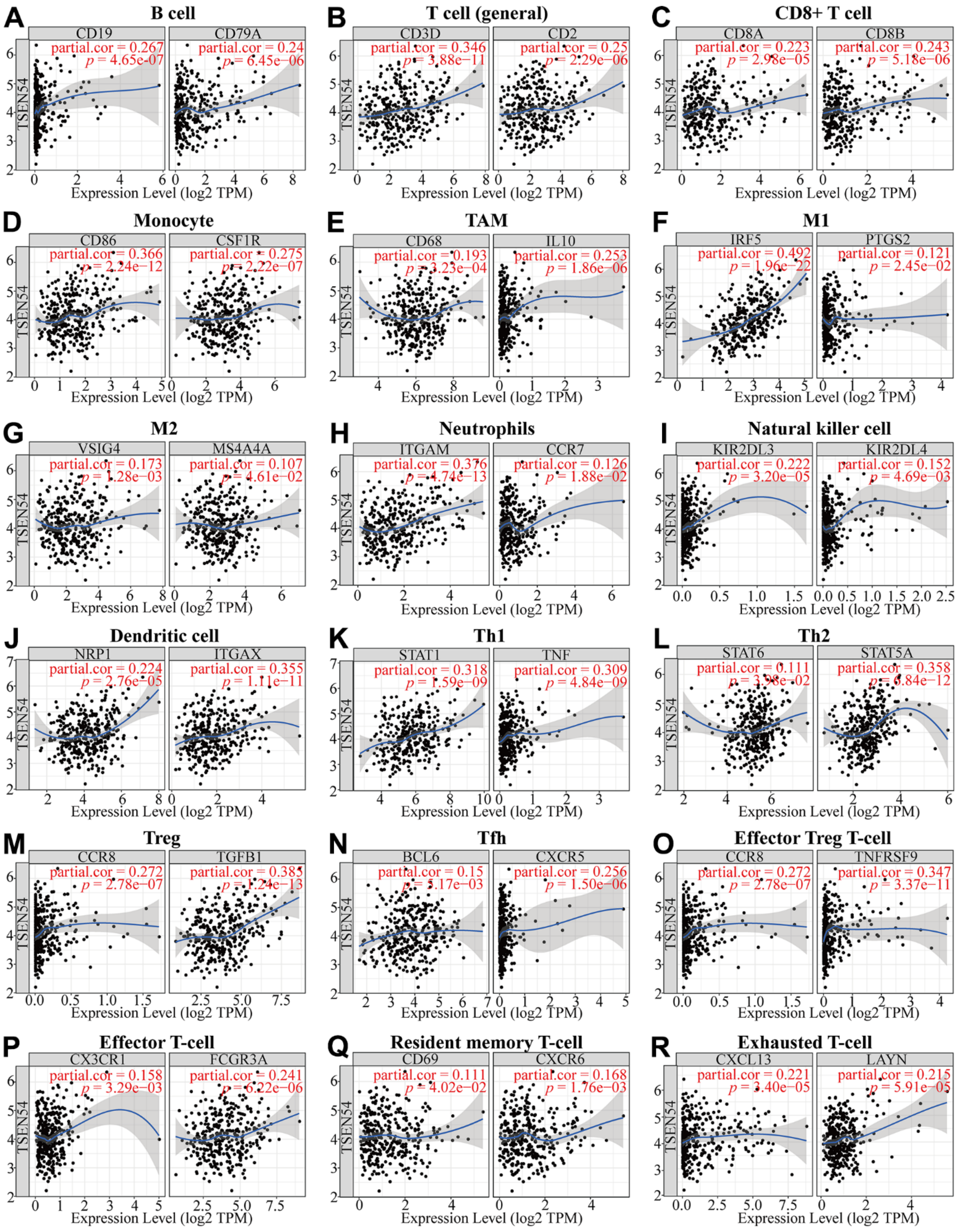

Supplementary Figure 2. Correlations between TSEN54 expression and immune cell markers in HCC. (**A**) B cell; (**B**) T cell (general); (**C**) CD8+ T cell; (**D**) Monocyte; (**E**) TAM; (**F**) M1; (**G**) M2; (**H**) Neutrophils; (**I**) Natural killer cell; (**J**) Dendritic cell; (**K**) Th1; (**L**) Th2; (**M**) Treg; (**N**) Tfh; (**O**) Effector Treg T-cell; (**P**) Effector T-cell; (**Q**) Resident memory T-cell; (**R**) Exhausted T-cell.

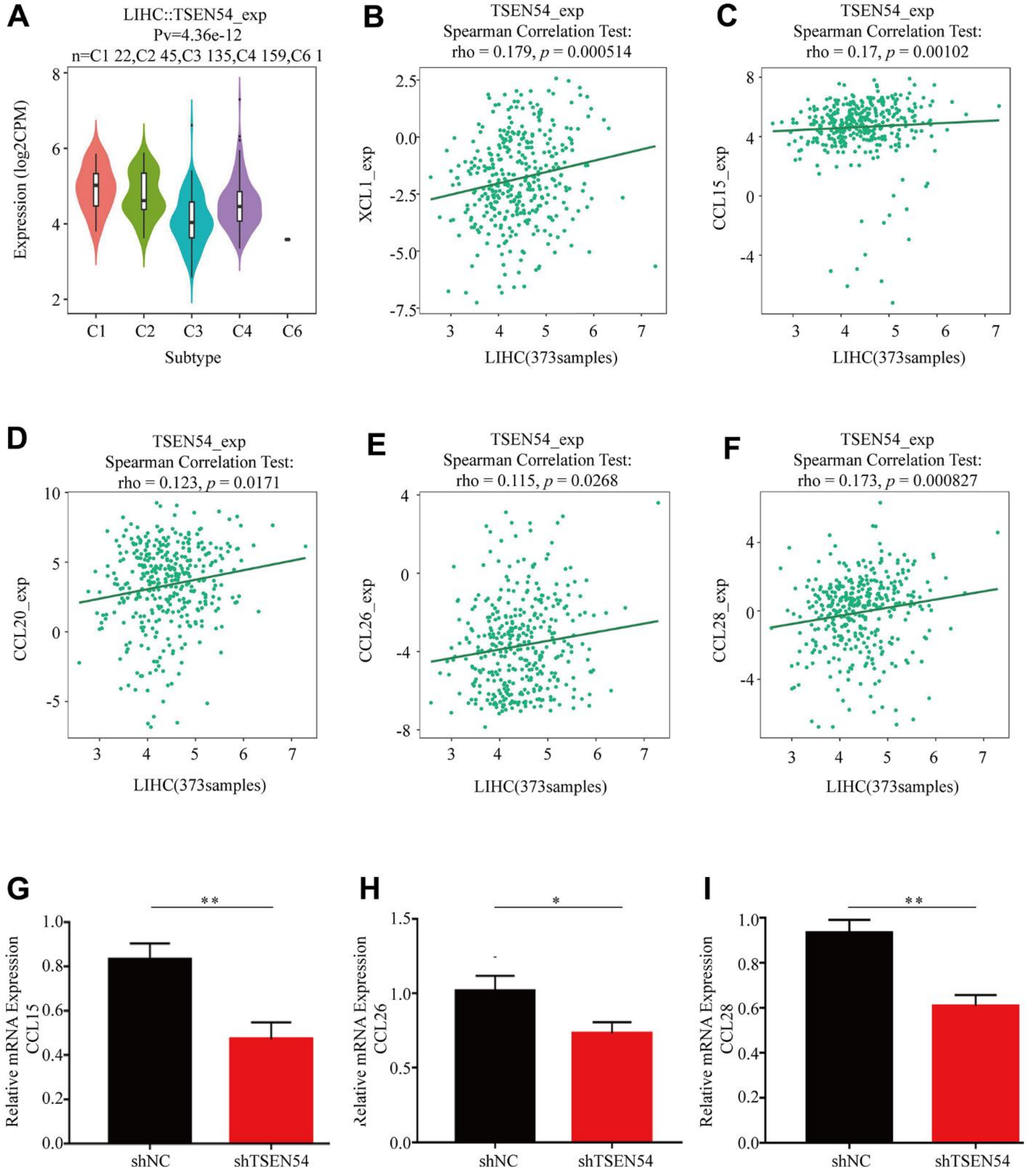

Supplementary Figure 3. Association of TSEN54 expression with immune subtypes and chemokines expression in hepatocellular carcinoma. (**A**) Expression of TSEN54 in different immune subtypes. Association between TSEN54 expression and expression of chemokine (**B**) XCL1; (**C**) CCL15; (**D**) CCL20; (**E**) CCL26; (**F**) CCL28. Expression levels of chemokines (**G**) CCL15; (**H**) CCL26; (**I**) CCL28 in HCCLM3 cells undergoing different treatments. *$p < 0.05$, **$p < 0.01$, ***$p < 0.001$.

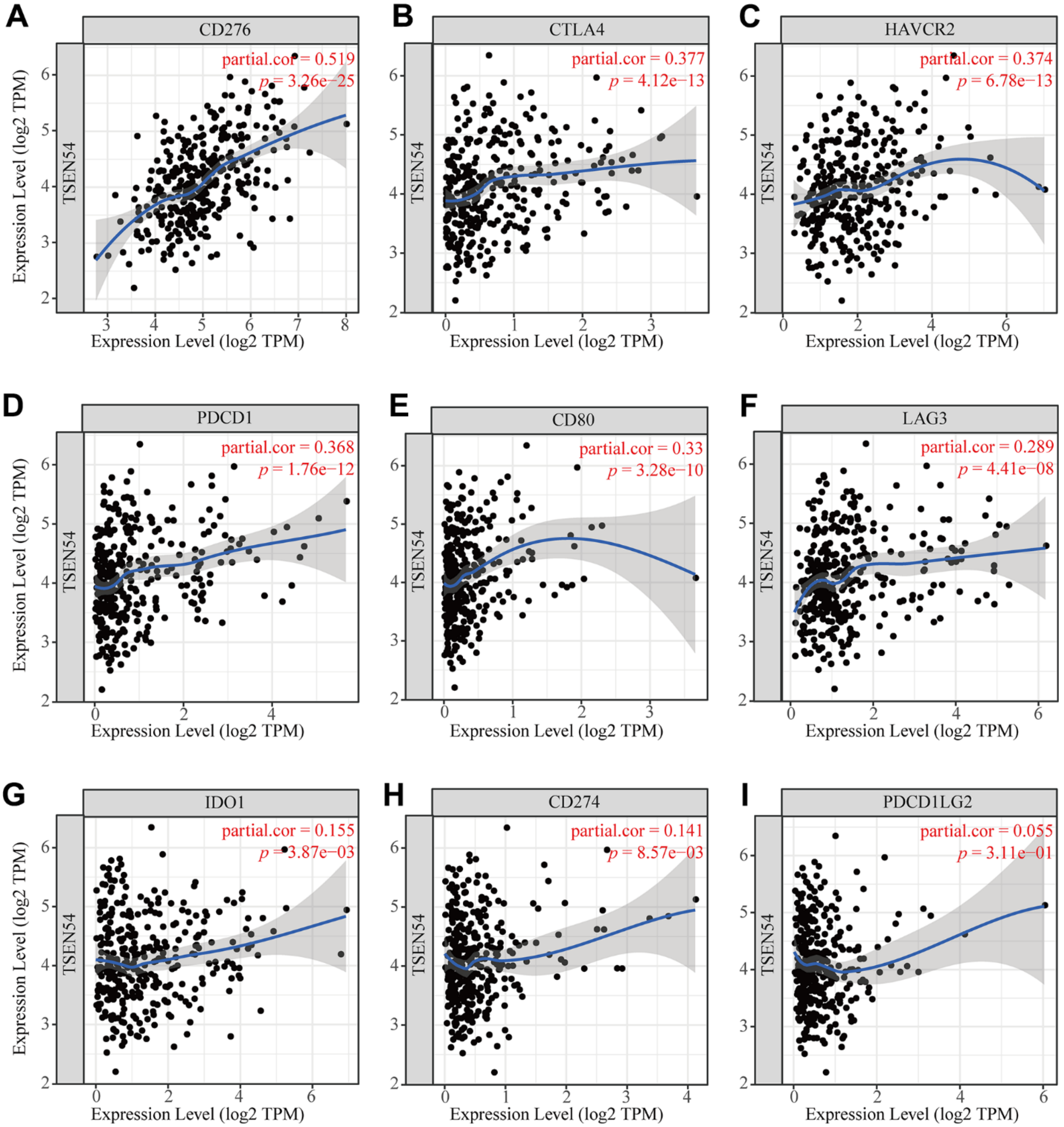

Supplementary Figure 4. Correlation between the expression level of TSEN54 and immune checkpoints in HCC. (**A**) CD276; (**B**) CTLA4; (**C**) HAVCR2; (**D**) PDCD1; (**E**) CD80; (**F**) LAG3; (**G**) IDO1; (**H**) CD274; (**I**) PDCD1LG2.
