# Supplementary material for: Comprehensive analysis reveals TSEN54 as a robust prognosis biomarker and promising immune-related therapeutic target for hepatocellular carcinoma
Source: Aging (Albany NY). 2023 Apr 8;15(7):2734–71. doi: 10.18632/aging.204645 (PMC10120902; doi:10.18632/aging.204645)
Supplement: Supplementary Tables [file aging-15-204645-s002.pdf]

SUPPLEMENTARY TABLES

Supplementary Table 1. Logistic analysis of the association between TSEN54 expression and clinical characteristics.

Clinical characteristics	Total (N)	Odds ratio in TSEN54 expression	P value
**Age** (>60 vs. ≤60)	370	0.72(0.48-1.09)	0.119
**Gender** (Female vs. Male)	371	1.32(0.86-2.05)	0.210
**Grade** (III vs. I)	366	3.48(1.80-6.90)	**<0.001**
**Stage** (II vs. I)	347	2.26(1.34- 3.86)	**0.002**
**T** (T2 vs. T1)	368	2.55(1.54-4.29)	**<0.001**
**M** (M1 vs. M0)	270	3.05(0.38-62.00)	0.338

T, tumor; M, metastasis; Bold values indicate P-values<0.05.

Supplementary Table 2. Univariate and multivariate COX regression analysis of factors associated with OS in liver cancer patients.

Variable	Univariate analysis			Multivariate analysis		
	HR	95%CI	P-value	HR	95%CI	P-value
**age**	1.007	0.990-1.024	0.441	1.007	0.990-1.024	0.404
**gender**	0.839	0.536-1.314	0.443	1.019	0.638-1.627	0.938
**grade**	1.073	0.795-1.449	0.645	1.158	0.838-1.599	0.375
**stage**	1.809	1.426-2.294	**<0.001**	1.053	0.420-2.638	0.913
**T**	1.767	1.415-2.207	**<0.001**	1.607	0.689-3.751	0.272
**M**	3.892	1.223-12.386	**0.021**	1.520	0.415-5.573	0.528
**TSEN54**	1.057	1.023-1.093	**0.001**	1.036	1.001-1.072	**0.042**

OS, overall survival; HR, hazard ratio; CI, confidence interval; T, tumor; N, node; M, metastasis; Bold values indicate P-values<0.05.

Supplementary Table 3. Correlation analysis between TSEN54 and gene markers of different types of immune cells in TIMER.

Description	Gene markers	LIHC			
		None		Purity	
		Cor	p	Cor	p
**B cell**	CD19	0.202389235	**8.64E-05**	0.267377717	**4.65E-07**
	CD79A	0.128911048	**0.012955142**	0.240176753	**6.45E-06**
**T cell (general)**	CD3D	0.234449883	**5.33E-06**	0.346004645	**3.88E-11**
	CD3E	0.105762836	**0.041791929**	0.241193771	**5.88E-06**
	CD2	0.124508092	**0.016468742**	0.251270165	**2.29E-06**
**CD8+ T cell**	CD8A	0.123889815	**0.017015243**	0.222738442	**2.98E-05**
	CD8B	0.146929914	**0.004569294**	0.242568159	**5.18E-06**
**Monocyte**	CD86	0.229956737	**8.11E-06**	0.366020432	**2.24E-12**
	CSF1R	0.147265925	**0.004509586**	0.274518328	**2.22E-07**
**TAM**	CCL2	0.076182565	0.142987697	0.171499465	**0.001385652**
	CD68	0.11589803	**0.025640279**	0.19251427	**0.000322508**
	IL10	0.150779612	**0.003601813**	0.253448179	**1.86E-06**
**M1**	IRF5	0.49114087	**6.38E-24**	0.492025997	**1.96E-22**
	PTGS2	0.021590413	0.678503783	0.121095345	**0.024489161**
**M2**	CD163	-0.037099129	0.476208689	0.044501381	0.409948133
	VSIG4	0.069820956	0.179530032	0.172752072	**0.001275952**
	MS4A4A	0.005913939	0.909570673	0.107434277	**0.046148153**
**Neutrophils**	CEACAM8	0.010704639	0.837184606	0.038274868	0.47857044
	ITGAM	0.295853945	**7.40E-09**	0.376340649	**4.74E-13**
	CCR7	0.015556345	0.765212213	0.126487372	**0.018757309**
**Natural killer cell**	KIR2DL1	-0.057048328	0.273072999	-0.088365409	0.101303959
	KIR2DL3	0.169935675	**0.001015924**	0.221890148	**3.20E-05**
	KIR2DL4	0.125536127	**0.015545474**	0.151887885	**0.004693128**
	KIR3DL1	-0.031109239	0.550287866	-0.025165954	0.641348507
	KIR3DL2	0.06023975	0.24709449	0.107992064	**0.045021948**
	KIR3DL3	0.043800893	0.400223792	0.044544858	0.409490419
**Dendritic cell**	HLA-DPB1	0.130829752	**0.011705036**	0.231304715	**1.43E-05**
	HLA-DQB1	0.102332112	**0.048920189**	0.188670792	**0.000426079**
	HLA-DRA	0.095978023	0.064810567	0.185502114	**0.000533893**
	HLA-DPA1	0.077766211	0.134843094	0.175633352	**0.001053238**
	CD1C	0.054657825	0.293710519	0.125151012	**0.020056474**
	NRP1	0.207734463	**5.74E-05**	0.223667441	**2.76E-05**
	ITGAX	0.241468544	**2.72E-06**	0.354940261	**1.11E-11**

Supplementary Table 4. Correlation analysis between TSEN54 and gene markers of different types of T cells in TIMER.

Description	Gene markers	LIHC			
		None		Purity	
		Cor	p	Cor	p
Th1	TBX21	0.008268494	0.873883393	0.092547169	0.086082606
	STAT4	0.13685461	**0.008343938**	0.183705472	**0.000605761**
	STAT1	0.279569814	**4.96E-08**	0.317637102	**1.59E-09**
	TNF	0.195364992	**0.000152569**	0.308501719	**4.84E-09**
	IFNG	0.214103055	**3.21E-05**	0.289851459	**4.20E-08**
Th1-like	HAVCR2	0.234246846	**5.44E-06**	0.373995183	**6.78E-13**
	IFNG	0.214103055	**3.21E-05**	0.289851459	**4.20E-08**
	CXCR3	0.181744948	**0.000443358**	0.288709772	**4.77E-08**
	BHLHE40	0.081995455	0.114842512	0.098127933	0.068694105
	CD4	0.065401128	0.208718979	0.133184303	**0.013291946**
Th2	STAT6	0.133242609	**0.010192215**	0.110737147	**0.039810208**
	STAT5A	0.314992604	**5.46E-10**	0.358338792	**6.84E-12**
Treg	FOXP3	0.013934159	0.789085628	0.062912231	0.243834173
	CCR8	0.188294211	**0.000265072**	0.272358282	**2.78E-07**
	TGFB1	0.280147907	**4.64E-08**	0.384976126	**1.24E-13**
Tfh	BCL6	0.145675934	**0.004966362**	0.150219762	**0.005174512**
	CXCR5	0.154108372	**0.002919221**	0.25562637	**1.50E-06**
Th17	STAT3	0.099431072	0.055716785	0.133663869	**0.012960765**
	IL17A	-0.021952393	0.67342148	-0.010627959	0.844066457
Resting Treg	FOXP3	0.013934159	0.789085628	0.062912231	0.243834173
	IL2RA	0.21861064	**2.16E-05**	0.337482259	**1.23E-10**
Effector Treg T-cell	FOXP3	0.013934159	0.789085628	0.062912231	0.243834173
	CCR8	0.188294211	**0.000265072**	0.272358282	**2.78E-07**
	TNFRSF9	0.268843967	**1.46E-07**	0.347024997	**3.37E-11**
Effector T-cell	CX3CR1	0.109856017	**0.034453452**	0.157832362	**0.003288359**
	FGFBP2	-0.166104568	**0.001323027**	-0.160474669	**0.00279651**
	FCGR3A	0.165879113	**0.001361374**	0.240568277	**6.22E-06**
Naïve T-cell	CCR7	0.015556345	0.765212213	0.126487372	**0.018757309**
	SELL	0.087163278	0.093649226	0.168112938	**0.00172701**
Effector memory T-cell	DUSP4	0.210204518	**4.66E-05**	0.310944533	**3.60E-09**
	GZMK	-0.030926455	0.552634182	0.072100061	0.181523666
	GZMA	0.050513824	0.33189635	0.142621342	**0.007977156**
Resident memory T-cell	CD69	0.012372955	0.812249658	0.110508722	**0.040223521**
	CXCR6	0.055378619	0.287380959	0.167829048	**0.001758867**
	MYADM	0.258235124	**5.04E-07**	0.300630413	**1.23E-08**
General Memory T-cell	CCR7	0.015556345	0.765212213	0.126487372	**0.018757309**
	SELL	0.087163278	0.093649226	0.168112938	**0.00172701**
	IL7R	-0.047784366	0.358718137	0.042168211	0.434952771
Exhausted T-cell	HAVCR2	0.234246846	**5.44E-06**	0.373995183	**6.78E-13**
	LAG3	0.245711889	**1.80E-06**	0.289404995	**4.41E-08**
	CXCL13	0.148664025	**0.004107682**	0.221172856	**3.40E-05**
	LAYN	0.156964964	**0.00245437**	0.214500211	**5.91E-05**
